# Intrinsic control of muscle attachment sites matching

**DOI:** 10.7554/eLife.57547

**Published:** 2020-07-24

**Authors:** Alexandre Carayon, Laetitia Bataillé, Gaëlle Lebreton, Laurence Dubois, Aurore Pelletier, Yannick Carrier, Antoine Wystrach, Alain Vincent, Jean-Louis Frendo

**Affiliations:** 1 Centre de Biologie du Développement (CBD)ToulouseFrance; 2 Centre de Recherche sur la Cognition Animale (CRCA)ToulouseFrance; 3 Centre de Biologie Intégrative (CBI), Université de Toulouse, CNRS, UPSToulouseFrance; National Centre for Biological Sciences, Tata Institute of Fundamental ResearchIndia; National Centre for Biological Sciences, Tata Institute of Fundamental ResearchIndia

**Keywords:** myology, transcription factors, muscle identity, *D. melanogaster*

## Abstract

Myogenesis is an evolutionarily conserved process. Little known, however, is how the morphology of each muscle is determined, such that movements relying upon contraction of many muscles are both precise and coordinated. Each *Drosophila* larval muscle is a single multinucleated fibre whose morphology reflects expression of distinctive identity Transcription Factors (iTFs). By deleting transcription cis-regulatory modules of one iTF, Collier, we generated viable muscle identity mutants, allowing live imaging and locomotion assays. We show that both selection of muscle attachment sites and muscle/muscle matching is intrinsic to muscle identity and requires transcriptional reprogramming of syncytial nuclei. Live-imaging shows that the staggered muscle pattern involves attraction to tendon cells and heterotypic muscle-muscle adhesion. Unbalance leads to formation of branched muscles, and this correlates with locomotor behavior deficit. Thus, engineering *Drosophila* muscle identity mutants allows to investigate, in vivo, physiological and mechanical properties of abnormal muscles.

## eLife digest

Each muscle in the body has a unique size, shape and set of attachment points. Animals need all of their muscles to have the correct identity to help maintain posture and control movement. A specific set of proteins, called transcription factors, co-ordinate and regulate gene activity in cells so that each muscle develops in the right way.

To create a muscle, multiple precursor cells fuse together to form a muscle fibre, which then elongates and attaches to specific sites. Correct attachment is critical so that the fibre is properly oriented. When this process goes wrong, for example in disease, muscle fibres sometimes attach to the wrong site; they become branched and cannot work properly.

Collier is a transcription factor protein that controls muscle identity in the fruit fly *Drosophila melanogaster*. However, like many transcription factors, Collier also has several other roles throughout the body. This made it difficult to evaluate the effect of the protein on the formation of specific muscles.

Here, Carayon et al. managed to selectively deactivate Collier in just one muscle per body section in the larvae of fruit flies. This showed that the transcription factor is needed throughout muscle development; in particular, it is required for muscle fibres to select the correct attachment sites, and to be properly oriented. Affected muscles showed an altered orientation, with branched fibres attaching to the wrong site. Even minor changes, which only affect a single muscle from each body segment, greatly impaired the movement of the larvae.

The work by Carayon et al. offers a new approach to the study of muscular conditions. Branched muscles are seen in severe human illnesses such as Duchenne muscular dystrophy. Studying the impact of these changes in a living animal could help to understand how this disease progress, and how it can be prevented.

## Introduction

The musculature of each animal is composed of an array of body wall muscles allowing precision and stereotypy of movements. The somatic musculature of the *Drosophila* larva - about 30 distinct body wall muscles per each hemi-segment which are distributed in three layers, internal, median and external - is a model to study how a muscle pattern is specified and linked to locomotion behaviour. Each muscle is a single multinucleated fibre with a specific identity: size, shape, orientation relative to the dorso-ventral and antero-posterior body axes, motoneuron innervation and attachment sites to the exoskeleton via tendon cells located at specific positions. Intersegmental tendon cells, where a large fraction of muscles is attached, are distributed in three groups, dorsal, lateral and ventral (Bate, 1990; Volk and VijayRaghavan, 1994; Schweitzer et al., 2010; Armand et al., 1994). Internal muscles also attach to muscle(s) in the next segment, forming ‘indirect’ muscle attachment sites (iMAS) (Maartens and Brown, 2015).

**Figure 1.**
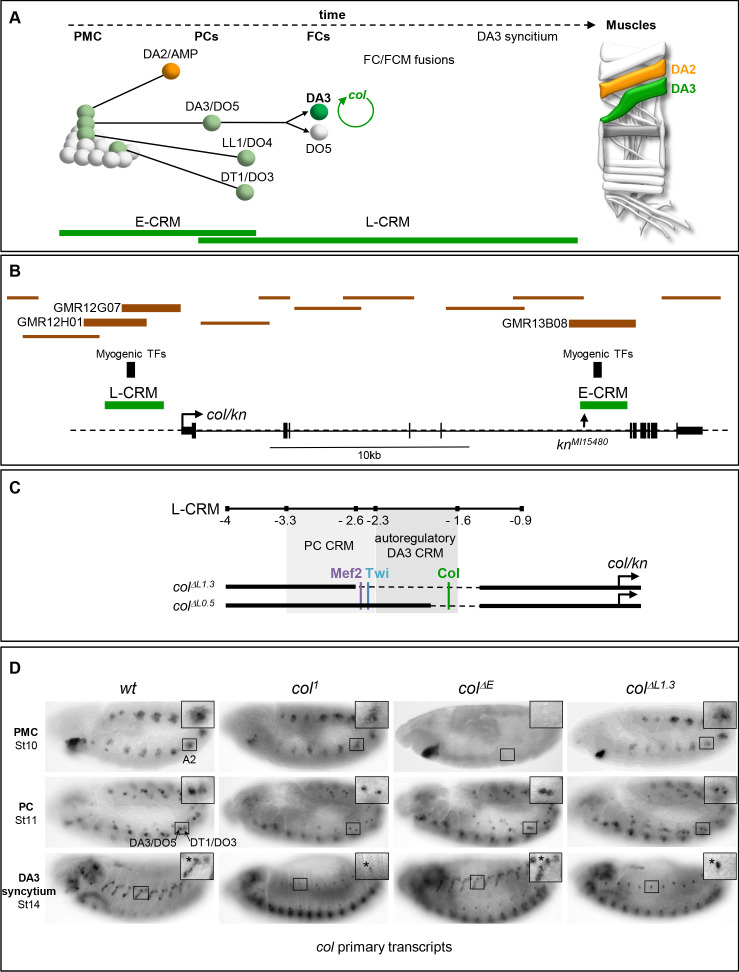
*Col* CRMs, CRM deletions and *col* transcription. (**A**) Diagrammatic representation of the sequential emergence of 4 different PCs from the Col-expressing PMC (grey), division of the DA3/DO5 PC into 2 FCs and *col* auto-regulation in the DA3 lineage; the names of each PC and FC are indicated. Accumulation of Col protein is in green. The time windows of mesodermal early (E-CRM) and late (L-CRM) CRM activity are indicated by green lines. Right: muscle pattern of an abdominal segment highlighting DA2 (orange) and DA3 (green). (**B**) Schematic representation of the *col* transcribed region (http://flybase.org/cgi-bin/gbrowse2/dmel/?Search=1;name=FBgn0001319). The position of tested GMR and VT fragments are drawn as brown horizontal bars; the numbers are given for those active in DA3. The positions of clusters of in vivo Mef-2, Twi and Tin binding sites are indicated by vertical blue bars, the *kn^Mi15480^* transposon insertion, used for the E-CRM deletion screening, by a vertical arrow. (**C**) Enlarged view of L-CRM indicating the juxtaposition of PC-specific and autoregulatory DA3-specific CRMs, Col, Mef2 and Twi binding sites, and the *col^ΔL1.3^* and *col^ΔL0.5^* deletions generated by CRISPR/cas9 genome editing. (**D**) *Col* transcription in wt and mutant embryos, genotypes indicated, visualised by in situ hybridisation to primary transcripts. A detail of the abdominal A2 segment (squared area) is shown in each panel. PMC *col* transcription is lost in *col^Δ^*^E^ embryos; stage 11, *col* transcription in the DA3/DO5 and DT1/DO4 PCs is detected in all strains; stage 14, DA3 syncytium transcription is lost in *col^1^* and *col ^ΔL1.3^* embryos. * indicates col transcription in a multidendritic neuron (md).

*Drosophila* muscle development proceeds through fusion of a founder myoblast (founder cell, FC) with fusion-competent myoblasts (FCMs) (Deng et al., 2017). FCs originate from asymmetric division of progenitor cells (PCs) themselves selected from equivalence groups of myoblasts, called promuscular clusters (PMCs). Muscle identity ensues from the expression by each PC and FC of a specific combination of identity transcription factors (iTFs) (de Joussineau et al., 2012), established in three steps: First, different iTFs are activated in different PMCs, in response to positional information, and this expression is only maintained in PCs. Second, refinement of the iTF code occurs via cross-regulations between different iTFs in PCs and/or FCs and Notch signalling (Carmena et al., 1998; Carmena et al., 2002; Enriquez et al., 2010). Several PCs can be serially selected from a PMC and give rise to different muscle identities according to birth order, adding a temporal dimension to these regulations. A well-documented example is the distinction between DA2 and DA3 identities (Figure 1A; Dubois et al., 2016; Boukhatmi et al., 2012). Third, transcriptional activation of iTFs in syncytial nuclei after fusion correlates with the activation of identity ‘realisation’ genes acting downstream of some iTFs (Crozatier and Vincent, 1999; Knirr et al., 1999; Bataillé et al., 2010; Bataillé et al., 2017).

**Figure 1—figure supplement 1.**
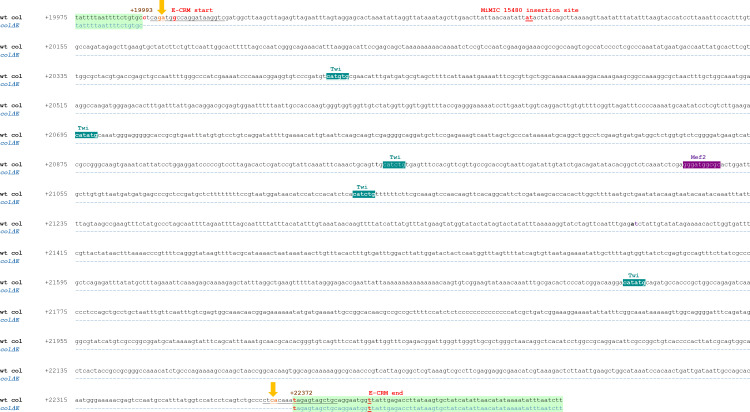
Genomic sequence of the E-CRM deletion. Genomic sequence spanning the previously mapped E-CRM (start and end indicated in red) and the *col ^ΔE^* deleted fragment (dashed blue line). The nucleotide positions of the *col ^ΔE^* deletion endpoints (brown) are given relative to the *col* transcription start site. The predicted cleavage sites of the used sgRNAs (sequences underlined) are indicated by yellow arrows. The MiMIC 15480 used for the screening of the deletion is inserted between the red underlined nucleotides. Predicted binding sites for Twist (blue) and Mef2 (purple), conserved between *Drosophila* species.

**Figure 1—figure supplement 2.**
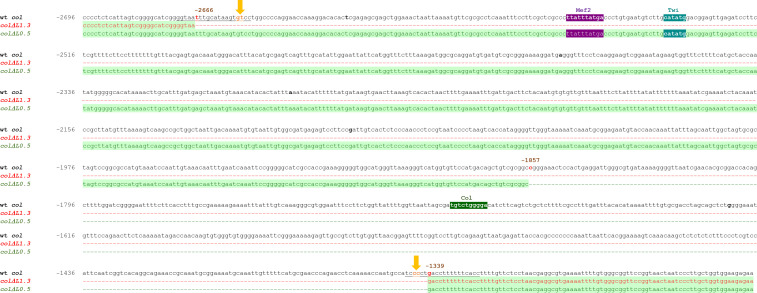
Genomic sequence of the L-CRM deletions. Genomic sequence spanning the *col ^ΔL1.3^* (from −2666 to −1339) and *col ^ΔL0.5^* (from −1857 to −1339) deletions. Deleted fragments are represented by dashed lines and their coordinates given relative to *col* transcription start site. The predicted cleavage sites of the used sgRNAs (sequences underlined) are indicated by yellow arrows. Predicted binding sites for Twist (blue), Mef2 (purple), and Col (green), conserved between *Drosophila* species.

**Figure 1—figure supplement 3.**
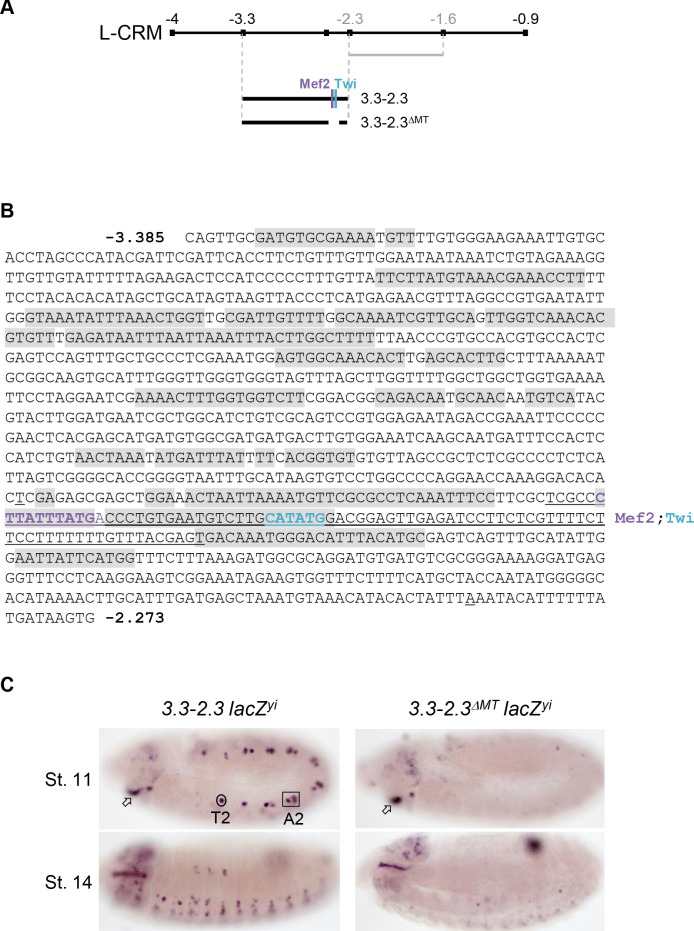
Mapping of a PC-specific CRM. (**A**) Schematic representation of *col* L-CRM (see also Figure 1) indicating the positions of the −2.3 to −1.6 fragment (grey) previously shown to drive expression in the DA3 syncytium and new reporter constructs, −3.3–2.3 and −3.3–2.3 ^ΔMT^ with the position of conserved Mef2 and Twi binding sites. (**B**) Nucleotide sequence of the −3.3–2.3 fragment, positions −3385 to −2273. The >20 bp sequence motifs that are more than 90% conserved at the same relative positions in *D. melanogaster*, *D. pseudobscura* and *D. virilis* are shaded in grey. The 97 bp segment containing both Mef2 (purple) and Twi (blue) binding and deleted in 3.3–2.3^ΔMT^ is underlined. (**C**) In situ hybridisation to primary reporter transcripts (*yellow* intron (*yi*) inserted in the *lacZ* coding sequence). The 3.3–2.3 fragment drives specific expression in the DA3/DO5 and DT1/DO4 PCs (circled in segment T2 and framed in A2) and the intercalary segment (arrow). Its activity in PCs is lost upon deletion of the region encompassing Mef2 and Twi binding sites (3.3–2.3^ΔMT^ reporter).

The consequence of specific muscle identity defects on locomotion, a question of prime importance for progress on studying human myopathies which affect subsets of muscles, remains largely to be assessed. Genetically controlled muscle identity changes should, in principle, provide suitable models for studying locomotion deficits linked to muscle imbalance. However, mutations for known *Drosophila* iTFs are embryonic lethal and/or show pleiotropic phenotypes reflecting their multiple expression sites. Here, we took advantage of our previous characterisation of *col* expression in a single larval muscle, the DA3 muscle and of the two involved cis-regulatory modules (CRMs), early (E-CRM) and late (L-CRM), to generate muscle-specific mutants and circumvent lethality/pleiotropy of null mutants.

CRM deletions show that E-CRM and L-CRM act redundantly at the PC stage, emphasising that PC is a key stage in specification of muscle identity. L-CRM deletion results into loss of *col* transcription in DA3 FCs and morphological transformation of the DA3 into a DA2-like muscle. Removal of an auto-regulatory cis-module located in L-CRM specifically abolishes *col* activation in syncytial nuclei fusing with the DA3 FC. This leads to incomplete DA3 transformations and the formation of bifid/branched muscles of mixed DA3/DA2 morphology. In summary, our data show that i) the FC transcriptional program must be propagated to syncytial nuclei for a muscle to adopt a specific morphology; ii) the precise matching of muscle/muscle attachments over the intersegmental border, which leads to a staggered rows pattern, involves a process of selective adhesion controlled by iTFs; iii) branched muscles affect larval locomotion performance.

Branched muscles are typical of late, severe phases of human Duchenne Muscular Dystrophy (Chan and Head, 2011). *Drosophila* iTF CRM deletion could be an effective setting for creating muscle-specific transformations and branched muscles, as new paradigms to study myopathies specifically affecting subsets of muscles in humans.

## Results

Redundant CRMs at the PC step ensure robustness of iTF transcription *col* transcription in myogenic lineages is first observed in a dorso-lateral PMC from which are sequentially selected several PCs (Dubois et al., 2016). It is subsequently maintained in two PCs, then one FC, the DA3 FC, and is activated in FCM nuclei recruited into the DA3 growing fibre (Crozatier and Vincent, 1999; Figure 1A). Two *col* CRMs containing embryonic in vivo binding sites for the master myogenic TFs, Mef-2 and Twist (Twi) (Sandmann et al., 2007; Zinzen et al., 2009) were previously identified, which reproduce this sequence of expression in reporter assays: E-CRM, which is active in the PMC and the DA3/DO5 and DT1/DO3 PCs, and L-CRM which is active in these same 2 PCs, the DA3 FC and DA3 syncytial nuclei (Enriquez et al., 2010; Dubois et al., 2007; Figure 1A; Figure 1—figure supplement 1 and Figure 1—figure supplement 2). With the prospect of using CRM deletions to generate muscle-specific mutants and to exclude the possible existence of additional, redundant enhancers (Cannavò et al., 2016), we conducted a systematic analysis of Gal4 reporter lines (Manning et al., 2012; Kvon et al., 2014) covering 36 kb of the *col* genomic region. A single reporter, GMR13B08, reproduces PMC Col expression and it overlaps E-CRM, and two reporters, GMR12G07 and GMR12H01, display DA3 expression and they both partly overlap L-CRM (Figure 1B and data not shown), attesting to the existence of only two *col* muscle CRMs.

The activity of E-CRM and L-CRM at different phases of muscle development raised the question of their respective roles in defining muscle identity. To address this question, we separately deleted each of them from the genome using the CrispR/Cas9 technology. Deletion of a 2,4 kb fragment removed the E-CRM. Two deletions within the L-CRM were generated: deletion of a 1.3 kb fragment removes the entire core region (Dubois et al., 2007) and deletion of a 0.5 kb fragment removes the Col autoregulation site (de Taffin et al., 2015; Figure 1C; Figure 1—figure supplement 1 and Figure 1—figure supplement 2). The corresponding mutant *Drosophila* strains are designated *col^ΔE^, col^ΔL1.3^* and *col^ΔL0.5^*, respectively. *col^ΔL^* strains are homozygous viable and fertile. *col^ΔE^* strain is homozygous female sterile, a sterility unrelated to *col* activity, since fertility is restored by placing *col^ΔE^* over a deficiency (*Df(2L)BSC429*) (abbreviated *Df* in the rest of the text and figures) removing the entire *col* locus (data not shown). As a first step to determine the consequences of deleting either E-CRM or L-CRM, we compared *col* transcription between wt, *col^1^* (a protein null mutant)*, col^ΔE^* and *col^ΔL1.3^* strains, using an intronic probe to detect nascent transcripts (Figure 1D). In *col^1^* homozygous mutant embryos, *col* transcription is detected at the PMC and PC stages and lost at the FC stage, showing the key role of autoregulation in the maintenance of col transcription (Crozatier and Vincent, 1999; de Taffin et al., 2015). In *col^ΔE^* embryos, we found that *col* is not activated in PMC cells, as could be expected. Yet, *col* transcription is detected in the DA3/DO5 and DT1/DO3 PCs, showing that inheritance of Col protein synthesised under E-CRM control is not required for activation of *col* transcription in PCs. A normal pattern is observed in the developing DA3 muscle, at stage 14. Reciprocal to E-CRM deletion, *col* is transcribed in PMC cells and the DA3/DO5 and DT1/DO3 PCs in *col^ΔL1.3^* embryos, but no more at stage 14, showing that L-CRM is required for *col* transcription maintenance.

Both E-CRM and L-CRM activity are detected in PCs. In absence of E-CRM, no Col protein is inherited from the PMC. Therefore, L-CRM activity in PCs cannot be due to *col* autoregulation. Analysis of new *col-lacZ^yi^* reporter genes (Perry et al., 2010) revealed the existence of a PC-specific CRM located within the −3.3 to −2.3 fragment of L-CRM, *i.e*., which is separate from the autoregulatory CRM defined by the in vivo Col binding site and contains the in vivo binding sites for Mef2 and Twist (Figure 1C; Figure 1—figure supplement 3; Zinzen et al., 2009). Activity of this PC-specific CRM is transient and lost upon removal of Mef2 and Twist binding sites (Figure 1—figure supplement 3). Overall, we conclude that two CRMs separately drive *col* transcription in muscle PCs, suggesting that robust iTF expression at the PC stage is critical to confer a muscle its identity.

### *col* CRM deletions lead to muscle transformation and branched muscles

**Figure 2.**
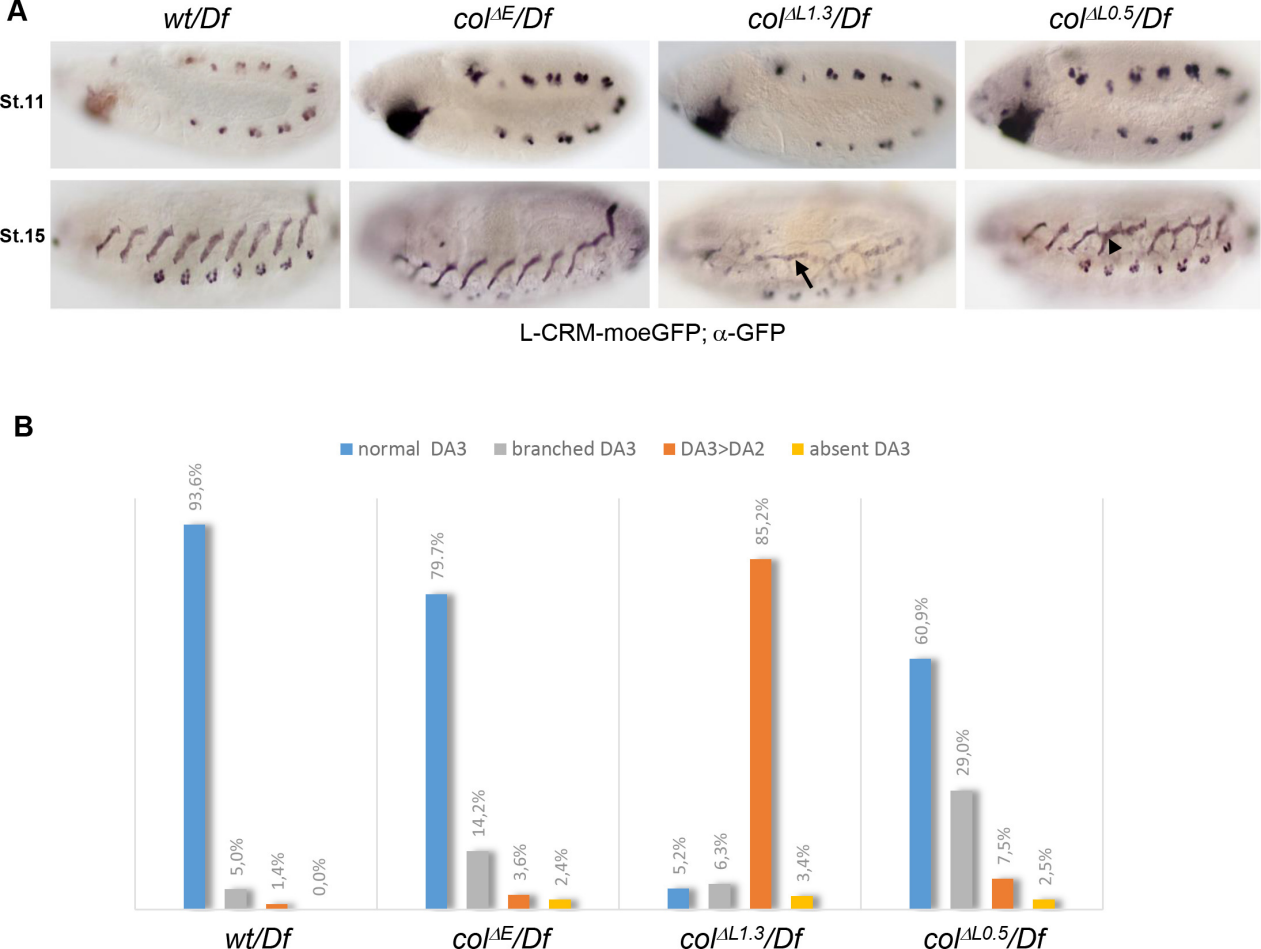
DA3 muscle transformations upon *col-CRM* deletions. (**A**) L-CRM-moeGFP expression in stage 11 and 15 hemizygous embryos, as indicated. GFP expression in PCs at stage 11 is similar in all strains. DA3^>DA2^ transformations (arrow) and branched DA3 muscles (arrowhead) are observed in *col^ΔL^* embryos. (**B**) Quantification of the relative proportions of normal DA3, branched DA3, DA3^>DA2^ transformation and absence of DA3 muscles in *wt*, *col^ΔE^*, *col^ΔL1.3^* and *col^ΔL0.5^* hemizygous embryos. A minimum of 100 A1-A7 abdominal segments of stage 15–16 embryos were analysed for each genotype. (+/Df: n = 127 segments - 16 embryos; *col^ΔE^*/Df: n = 170 segments - 23 embryos; *col^ΔL1.3^*/Df: n = 190 segments - 27 embryos; *col^ΔL0.5^*/Df: n = 103 segments - 13 embryos).

Having deleted separately each *col* CRM allowed to assess the respective roles of iTF transcription before or after the PC step. To compare the different CRM deletions, we placed each of them over the deficiency Df chromosome. We introduced the L-CRM-moeGFP reporter to visualise the DA3 morphology at stage 15 (Enriquez et al., 2012). Control (+/*Df*) embryos display reporter expression in the DA3/DO5 and DT1/DO3 PCs at stage 11 and the DA3 muscle at stage 15 (Figure 2A). The same pattern is observed in PCs for all *col*-CRM deletion strains, consistent with transcript analyses (Figure 1D). L-CRM-moeGFP expression at stage 15 shows that the DA3 morphology is normal in about 80% of segments in *col^ΔE^*/*Df* embryos (Figure 2A–B), and we did not pursue the analysis of this deletion strain. Low level GFP expression in *col^ΔL1.3^*/*Df* embryos, consistent with *col* transcription data (Figure 1D), shows that the DA3 muscle is most often (85.2% of segments) transformed into a DA2-like muscle (designated below as DA3^>DA2^; Figure 2A–B), like in *col* null mutant embryos (Enriquez et al., 2012). In *col^ΔL0.5^*/*Df* embryos, *i.e*, when only the autoregulation module has been deleted, a high number (29%) of branched muscles is observed (Figure 2A–B). Branched muscles correspond to incomplete transformations, with two stable anterior attachment sites, overlapping the DA3 and DA2 sites in *wt* embryos. The high ratios of either complete (DA3^>DA2^) or incomplete (branched) transformations in L-CRM deletion mutants demonstrate that an iTF CRM deletion strategy is effective for creating viable muscle-specific identity mutants and explore branched muscle properties.

### Re-programming of syncytial nuclei is required for muscle morphological identity

**Figure 3.**
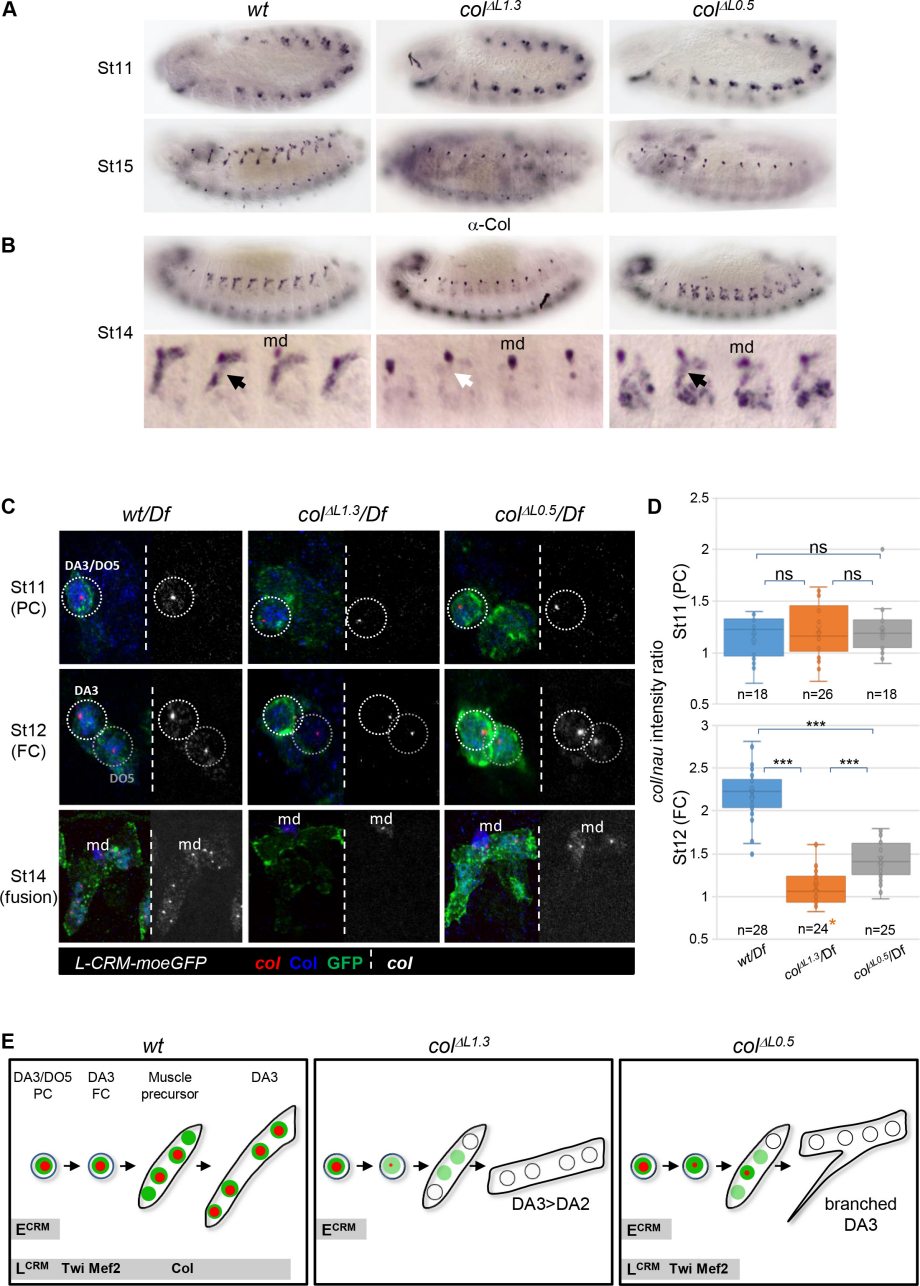
Identity reprogramming of syncytial nuclei controls the final muscle morphology. (**A**) Col immunostaining of wt, *col^ΔL1.3^* and *col^ΔL0.5^* embryos, showing a normal pattern at stage 11 and complete absence of Col protein at stage 15 in both *col^ΔL^* embryos. (**B**) Stage14 embryos with a close up view of 4 segments shows low amounts of Col protein (black arrow) in the growing DA3 muscle in *col^ΔL0.5^* and absence in *col ^ΔL1.3^* embryos (white arrow). Position of the multidendritic neuron (md) is indicated. (**C**) *Col* transcription (red dots), Col protein (blue) and L-CRM-moeGFP expression (green) in the DA3/DO5 PC, stage 11, DA3 FC, stage 12, and developing DA3 muscle, stage 14, in *wt/Df*, *col^ΔL1.3^/Df* and *col^ΔL0.5^/Df*; L-CRM-moeGFP embryos. In each panel, *col* transcripts are shown separately in black and white. *col* transcription ceases after FC stage in *col^ΔL1.3^* embryos and does not propagate to other syncytial nuclei in *col ^ΔL0.5^* embryos. (**D**) Quantification of col primary transcripts level in PC and FC nuclei; orange asterisk: *col* transcription in FCs is generally lost in *col^ΔL1.3^* embryos; quantification was done on a small fraction of FCs; n: number of PC or FC analysed, using 5 or six embryos at each stage 11 and 12; 15 *col^ΔL1.3^* embryos were used for the FC stage (Mean ± sem and ***: p<0.001). (**E**) Schematic representation of the dynamics of *col* transcription (red dots) and Col protein (green) in the DA3/DO5 PC, the DA3 FC, muscle precursor, and DA3, DA3^>DA2^ and branched DA3 muscles in wt, *col^Δ1.3^* and *col ^ΔL0.5^* embryos, respectively. Temporal activity of E-CRM and L-CRM is represented by horizontal grey bars.

Complete *vs* incomplete transformations in *col^ΔL1.3^* versus *col^ΔL0.5^* deletions suggest that proper level and/or maintenance of iTF expression is crucial for proper muscle development. This led us to compare the pattern of Col protein in growing DA3 syncytium between *wt*, *col^ΔL1.3^* and *col^ΔL0.5^* embryos. In either deletion strain, Col is detected in PCs at stage 11 but not in muscles at stage 15 (Figure 3A). However, at stage 14, some Col protein is still detected in muscle precursors in *col^ΔL0.5^*, not in *col^ΔL1.3^* embryos (Figure 3B). To trace the origin of this difference, we examined *col* transcription in the DA3 PC, FC and stage 14 syncytium using *Df/*hemizygous embryos which display one hybridisation dot per active nucleus (Figure 3C). In control *wt/Df* embryos, a dot is systematically detected in the DA3/DO5 PC (20/20 segments; five embryos analysed), the DA3 FC (20/20) and 80% of DA3 nuclei at stage 14 (6 of 7–8 nuclei per fibre on average; 27 segments). A dot is detected as well in the DA3/DO5 PC, in either *col^ΔL1.3^* (21/21) or *col^ΔL0.5^* (18/18) embryos, reflecting E-CRM activity (Figure 1D). In *col^ΔL0.5^* embryos, a *col* hybridisation dot is detected in the DA3 FC (19/19) and in one nucleus, likely the FC nucleus (11/21 segments), sometimes two nuclei at stage 14. In *col^ΔL1.3^* embryos, however, *col* transcription is only detected in a minor fraction of FCs (4/15) and is completely lost at stage 14 (0/6–7 nuclei per fibre on average; 24 segments). Patterns similar to stage 14 are observed at stage 15, while at stage 16, *col* transcription is detected neither in L-CRM deletion strains nor in control (Figure 3—figure supplement 1). Since *col* transcription at, and from, the PC stage appears to be nodal to DA3 identity, we measured the level of *col* transcripts relative to *nautilus* (*nau*), the *Drosophila MyoD-MRF* serving as internal reference (Figure 3D and Figure 3—figure supplement 2). As expected, similar levels of *col* transcription are found in the DA3 PC, in control (Mean ± sem: 1.22 ± 0.06; n = 18), *col^ΔL1.3^* (1.15 ± 0.04; n = 26) and *col^ΔL0.5^* embryos (1.19 ± 0.04; n = 18), which confirms handling by the E-CRM, with only a minor contribution from the L-CRM (Figure 1D). On the contrary, high level of *col* transcription in the DA3 FC in wt embryos (2.20 ± 0.05; n = 28) is dependent upon L-CRM activity, since a drop is observed in *col^ΔL1.3^* FCs (1.10 ± 0.04; n = 24), p<0.001. More precisely, it is dependent upon the presence of Mef2 and Twist binding sites since a basal level of transcription is still observed when only the *col* autoregulation module is deleted (*col^ΔL0.5^*: 1.41 ± 0.04; n = 25). Quantification of *col* and *nau* transcripts shows that the *col/nau* increase between the PC and FC stages in wt embryo is due to increased *col* transcription while the level of *nau* is relatively constant and is unaffected in *col* L-CRM mutants (Figure 3—figure supplement 2). Together, the data suggest that binding of Mef2 and Twist is required to prime *col* transcription in the FC nucleus before autoregulation takes off (Figure 3E). Taken with one another, Col immunostaining, FISH data and *col* transcription quantification indicate that sustained *col* transcription in the FC nucleus in *col^ΔL0.5^* embryos (Figure 3C–D), provides enough Col protein for some uptake by other DA3 nuclei at stage 14, and this leads to their partial reprogramming to DA3 identity. Partial reprogramming could, in turn, explain the formation of branched muscles retaining some DA3 morphological characters. Moreover, the *col^ΔL1.3^* and *col^ΔL0.5^* expression data and deletion phenotypes show that both iTF transcription in the FC and reprogramming of ‘naïve’ syncytial nuclei contribute to ensure robust muscle morphological identity (Figure 3E).

**Figure 3—figure supplement 1.**
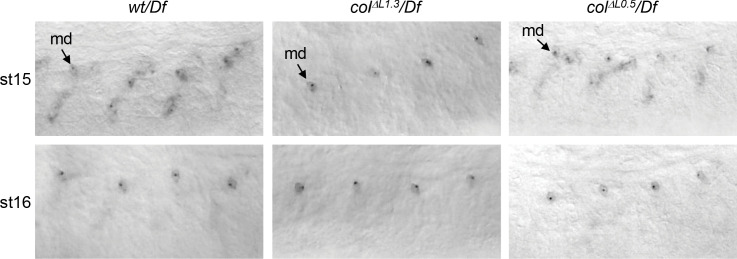
Col transcription in *col^ΔL0.5^* and *col^ΔL1.3^* embryos In situ hybridisation to *col* primary transcripts in stage 15 and 16 *wt/Df*, *col^ΔL1.3^/Df* and *col^ΔL0.5^/Df* embryos. At stage 15, dots corresponding to *col* transcription are detected in syncytial DA3 nuclei in *wt* and *col^ΔL0.5^/Df* embryos. At stage 16, *col* transcription is no more detected neither in control nor in L-CRM deletions, except in multidendritic (md) ddaC neurons, indicated by an arrow.

**Figure 3—figure supplement 2.**
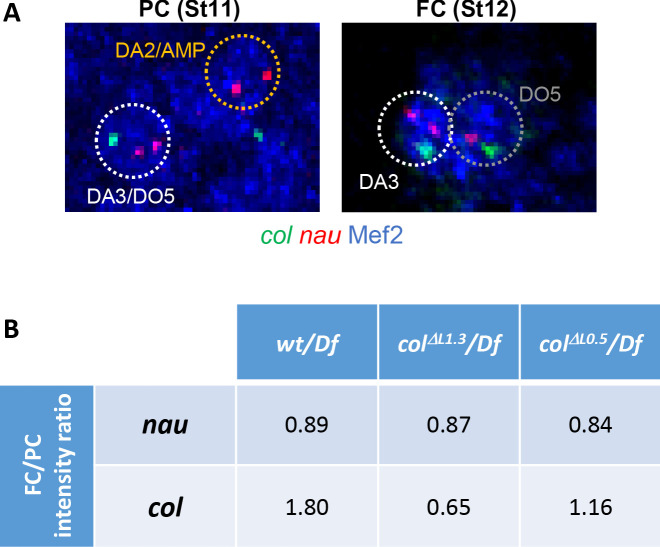
*Nau* expression is not affected in *col^ΔL0.5^* and *col^ΔL1.3^* embryos. (**A**) DA3 *col* (green) and *nau* (red) transcription dots at the PC and FC stage. Nuclei are labelled by Mef2 immunostaining (blue) and ROIs (regions of interest) used for quantification are circled. One *col* and two *nau* dots are observed in heterozygous (*wt/Df*, *col^ΔL1.3^/Df* and *col^ΔL0.5^/Df*) embryos. (**B**) Calculated dot intensity ratios (mean intensity) between the FC and PC stages. Number of nuclei used to calculate the mean dot intensity: *nau:* wt PC n = 28, FC n = 19; *col^ΔL1.3^* PC n = 24, FC n = 26; *col^ΔL0.5^* PC n = 25, FC n = 18; for *col*: wt PC n = 19, FC n = 28; *col^ΔL1.3^* PC n = 26, FC n = 25; *col^ΔL0.5^* PC n = 18, FC n = 24. *nau* data indicate a slight decrease between the PC and FC stages in all three conditions. *col* data show increased transcription between the PC and FC stage in wt embryos.

### Muscle attachment: tendon attraction and muscle-muscle matching

**Figure 4.**
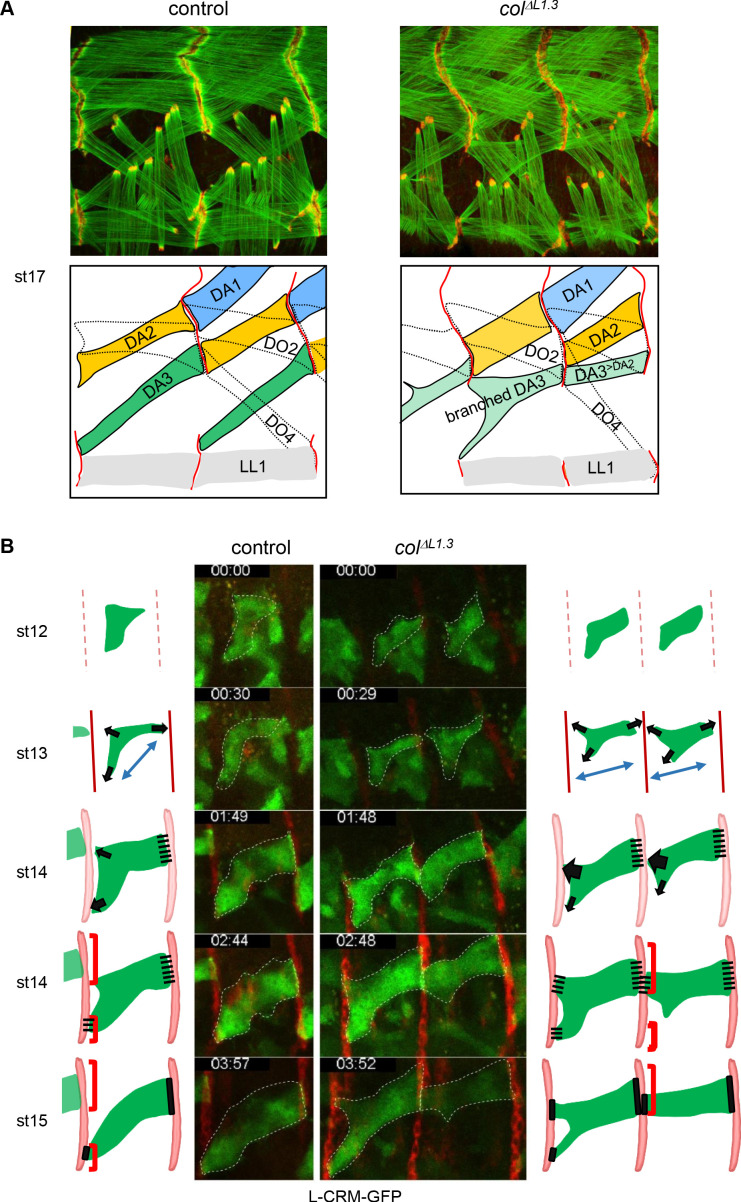
Multistep muscle attachment to selected tendon cells. (**A**) F-actin (green) and Ilk-GFP (red) staining of stage 17 control and *col^ΔL1.3^* embryos, showing heterotypic and homotypic muscle attachment sites, respectively. A drawing illustrates muscle matching in control embryos and mismatching in *col ^ΔL1.3^* embryos, with DA1, DA2 and DA3 muscles coloured in blue, yellow and green, respectively and attachment sites as red lines. (**B**) Snapshots of live imaging DA3 muscle development, using L-CRM-moeGFP expression (green). Tendon cell precursors express stripe-Gal4; UAS-RFP (red). Embryos were filmed during 4 hr (Sup. Videos 1 and 2) and Z sections collected every ~2–2.5 min. The outlines of the developing muscles are schematised for each stage. In both control (left, one segment shown) and *col^ΔL1.3^* embryos (right, two segments shown), the posterior muscle end reaches the intersegmental border first. At stage 14, *wt* DA3 contacts both dorsal and lateral tendon cells at its anterior end. In *col^ΔL1.3^* embryos, the anterior dorsal projections fail to retract, leading to ectopic DA3^>DA2^ attachment and branched muscles.

We next investigated in more detail how ectopic muscle attachment sites at the origin of transformed and branched muscles are selected and stabilised. Double staining of ready-to-hatch (st17) *wt* embryos for F-actin and Ilk-GFP, a component of muscle attachment sites (Zervas et al., 2011; Sarov et al., 2016) shows that the DA3 posterior edge anchors to dorsal, and anterior edge to lateral intersegmental tendon cells (Figure 4A), giving its final acute shape (Bate, 1990). Moreover, the DA3 and DA2, as well as the DA2 and DA1 muscles precisely align with each other over each intersegmental border, forming heterotypic muscle-muscle attachment (iMAS; [Maartens and Brown, 2015]) at the origin of the staggered rows disposition of DA muscles. No DA3/DA3 (homotypic) iMAS surface is observed (Figure 4A). On the contrary, in *col^ΔL1.3^* embryos, the anterior edge of DA3^>DA2^ transformed muscles anchors to dorsal tendon cells instead of lateral intersegmental tendon cells, leading to a ‘dual DA3^>DA2^ morphology’, DA2-like at the anterior, and DA3 at the posterior edge. This dual identity leads to iMASs between adjacent DA3^>DA2^ muscles (Figure 4A). As a consequence, DA2 iMASs are shifted dorsally and the general pattern of DA muscles is affected.

To explore the dynamics of DA3 muscle attachment, we live-imaged *wt* and *col^ΔL1.3^* embryos, starting at stage 12 (defined as t_0_ in Videos 1 and 2). The DA3 muscle is visualised by L-CRM-moeGFP and tendon cell along the entire intersegmental border by *stripe* Gal4;UASmCD8RFP (Volohonsky et al., 2007). In *wt* embryos between stages 12 and 13 (Video 1; Figure 4B), the DA3 muscle precursor extends protrusions towards dorsal tendon cells, posteriorly, and both dorsal and lateral tendon cells, anteriorly, diverging from a bipolar extension scheme (Schnorrer and Dickson, 2004). At stage 14, the posterior DA3 edge makes contacts with dorsal tendon cells before the anterior edge(s) reaches the intersegmental border, a time gap previously observed during live-imaging of a ventro-lateral muscle (Gilsohn and Volk, 2010). In addition, numerous filopodia emanate from the DA3 dorsal surface and contact the DA2 ventral surface, contributing to give the DA3 muscle precursor its transient angled-shape. At late stage 14, the posterior DA3 attachment widens parallel to the intersegmental border. Its anterior attachment to lateral tendon cells in turn becomes stable, whereas dorsal anterior filipodia appear to be repelled from the intersegmental border where the DA3 muscle in the preceding segment is attached. Remaining filopodia are limited to the rims of DA3 anchoring, possibly suggesting a mechanism of homotypic repulsion. In *col^ΔL1.3^* embryos, the main axis of the DA3^>DA2^ muscle precursor elongation is more longitudinal than *wt*, already at stage 12 (Video 2; Figure 4B). At stage 13 and like in *wt*, many protrusions emanate from the DA3 dorsal surface, but unlike in *wt*, protrusions contacting dorsal tendon cells do not retract at later stages when they contact the DA3^>DA2^ muscle from the preceding segment. Rather, stabilisation of contacts made by these protrusions prefigures abnormal DA3^>DA2^/DA3^>DA2^ iMAS formation (Figure 4A). In some segments, contacts with lateral tendon cells are also stabilised, resulting into branched muscles. In summary, imaging of live *wt* embryos illustrates different steps involved in establishment of the acute DA3 orientation: posterior attachment to dorsal tendon cells at the same time as anterior exploration of dorsal and lateral tendon cell. This is followed by DA3 attachment to lateral, and retraction from dorsal tendon cells (Figure 4B, Video 1), cumulating into heterotypic DA3/DA2 iMAS stabilisation (Figure 4A and B). This retraction does not take place in *col^ΔL1.3^* embryos (Figure 4B and Video 2).

**Video 1.** Live-imaging of *wt* embryos. The DA3 muscle is visualised by L-CRM-moeGFP (green) and tendon cell along the entire intersegmental border by *stripe* Gal4;UASmCD8RFP (red). Embryos were filmed during 4 hr. See also the legend of Figure 4.

**Video 2.** Live-imaging of *col^ΔL1.3^* embryos. The DA3 muscle is visualised by L-CRM-moeGFP (green) and tendon cell along the entire intersegmental border by *stripe* Gal4;UASmCD8RFP (red). Embryos were filmed during 4 hr. See also the legend of Figure 4.

### Muscle attachment: heterotypic adhesion

**Figure 5.**
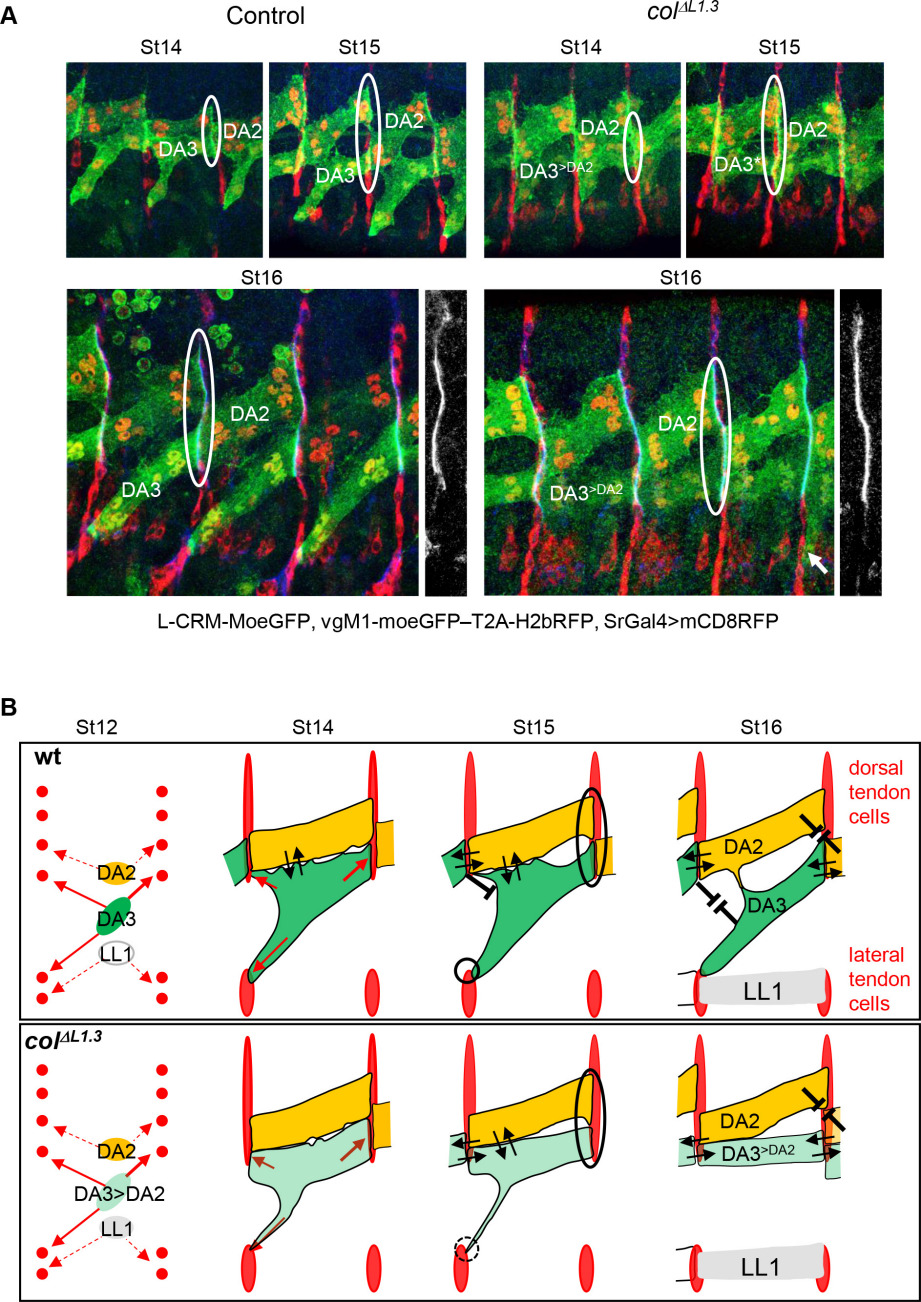
DA3 heterotypic and homotypic muscle attachment sites (iMAS) in wt and L-CRM mutants. (**A**) Immunostaining of *wt* and *col^ΔL1.3^* VgM1-moeGFP-H2bRFP; srGal4 >mcd8 RFP; L-CRM-moeGFP embryos for GFP (green), RFP (red) and Spectrin (blue). DA3 (DA3^>DA2^) and DA2 muscles are indicated in each panel. At stage 14, the *wt* DA3 and *col^ΔL1.3^* DA3^>DA2^ attachment sites partly align over the intersegmental border (circled). Their dorsal surface closely contacts DA2 *via* numerous protrusions; at stage 15, likely because homotypic DA3/DA3 and DA2/DA2 repulsion, these protrusions are restricted away from intersegmental borders in *wt* embryos while maintained in *col^ΔL1.3^*. At stage 16, stable heterotypic DA3/DA2 contacts in *wt*, and both homotypic DA3^>DA2^/DA3^>DA2^ and DA3^>DA2^/DA2 contacts are stabilised. Anti-Spectrin staining is shown in black and white on the right of each panel, to indicate the position of the dorsal iMASs. (**B**) Schematic drawings of DA muscle development (green) in *wt* and *col^ΔL1.3^*embryos; interpreted from data in Figure 4 and supplementary videos. Arrows indicate attractive cues, broken lines repulsive cues, thin circles attachment initiation, thick circles, attachment stabilisation. DA3 MAS initiation starts earlier at its posterior than anterior end. Homotypic repulsion between muscles of same identity leads to DA3 posterior attachment to lateral tendon cells. Repulsion does not operate upon DA3^>DA2^ transformation, leading to stable homotypic DA3^>DA2^ and DA3^>DA2^/DA2 iMASs.

Further determining how DA3^>DA2^/DA3^>DA2^ homotypic attachment could interfere with the staggered rows pattern of DA muscles, required to visualise at the same time the DA3 and DA2 muscles and tendon cells contours. Vestigial (Vg) is expressed in DA muscles (Deng et al., 2010; Tixier et al., 2010). We screened the *vg* regulatory landscape and characterised one *vg* CRM active in the DA3 and DA2 (and VL1) muscles, VgM1 (Figure 5—figure supplement 1). Expressing together VgM1-mCD8GFP-H2bRFP, L-CRM-moeGFP and stripeGal4:UAS-mCD8RFP, and co-staining of stage 16 control embryos for α-Spectrin, a protein enriched at muscle attachment sites, shows that the posterior edge of DA3 precisely aligns with the anterior edge of DA2 (Figure 5A, Figure 5—figure supplement 1), suggesting heterotypic attractive cues. This attraction is already observed at stage 14, when the dorsal DA3 and ventral DA2 surfaces contact each other *via* numerous protrusions (Figure 5A and B; Figure 5—figure supplement 2). Strikingly, at stage 15, the contact zone gets away from intersegmental borders and the DA3/DA3 and DA2/DA2 homotypic connections disappear, to give way to heterotypic DA3/DA2 stable iMAS formation. In *col^ΔL1.3^* embryos, the initial steps, stages 14 and 15, are similar to *wt*, except for the absence of stable connection of DA3^>DA2^ to lateral tendon cells, as seen by live imaging (Video 2). At stage 16, DA3^>DA2^/DA3^>DA2^ and DA3^>DA2^/DA2 iMASs are privileged, while similar to control, no homotypic DA2/DA2 iMAS forms, suggesting homotypic repulsion (Figure 4; Figure 5A). Together, the data indicate that the precise pattern of iMASs and muscles is contributed by a combination of attractive and, possibly, repulsive cues downstream of muscle iTFs (Figure 5B).

**Figure 5—figure supplement 1.**
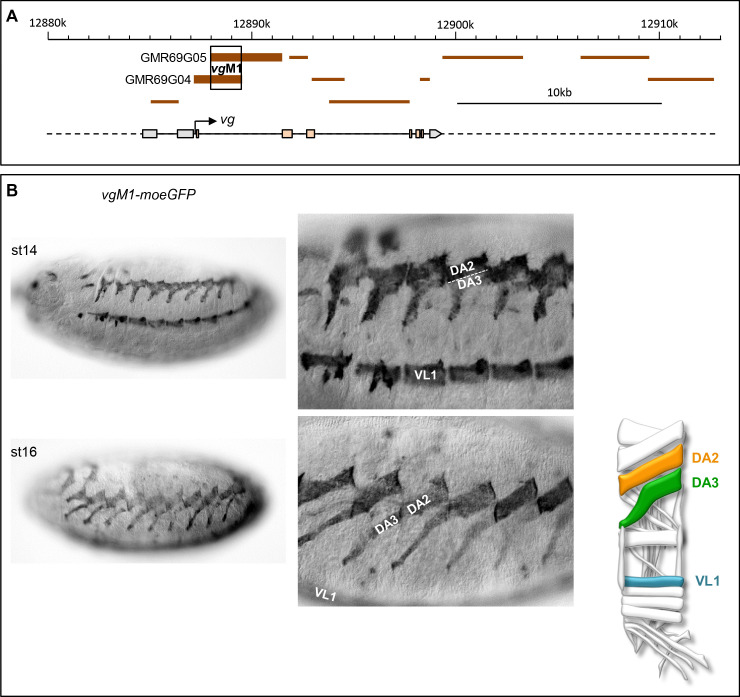
Identification of a *vg* CRM active in the DA2 and DA3 muscles. (**A**) Schematic representation of the *vestigial* (*vg*) transcribed region (https://flybase.org/cgi-bin/gbrowse2/dmel/?Search=1;name=FBgn0003975). GMR fragments tested for their enhancer activity are drawn as brown horizontal bars; GMR69G04 and GMR69G05 are active in DA3 and DA2 muscles. The fragment of overlap between GMR69G04 and GMR69G05, positions 12889557–12891520 on Chr2R, is called *vgM1* (**B**) *vgM1*-moeGFP expression in the DA2, DA3 and VL1 muscles in wild type embryos at stages 14 and 16 (schematised on the right); higher magnification views show the close apposition of the DA3 ventral and DA2 dorsal sufaces at stage 14 and their tandem arrangement at stage 16. Of note, vgM1 expression seems to be more robust in DA2 than DA3.

**Figure 5—figure supplement 2.**
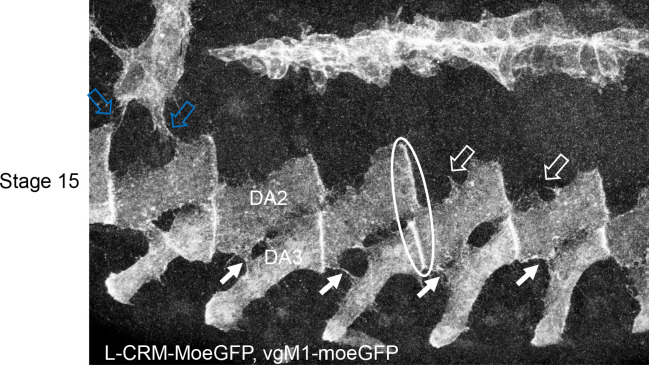
Interactions between the developing DA2 and DA3 muscles via dynamic protrusions Immunostaining of a stage 15 VgM1-moeGFP-H2bRFP; srGal4 >mcd8 RFP; L-CRM-moeGFP embryo. Only the GFP channel is shown, in black and white, to highlight protrusions emanating from the DA3 and DA2 muscles which contact each other (white arrows). Similar protrusions are seen between the DA2 and DA1 muscle (open arrows) which expresses VgM1-moeGFP in the thoracic T2 muscle (blue open arrows). The DA3/DA2 and DA2/DA1 muscle-muscle attachments are circled in white at one segment boundary. The observed protrusions are very dynamic as observed in videos and schematised in Figure 5B.

### Muscle strength lines in larvae

**Figure 6.**
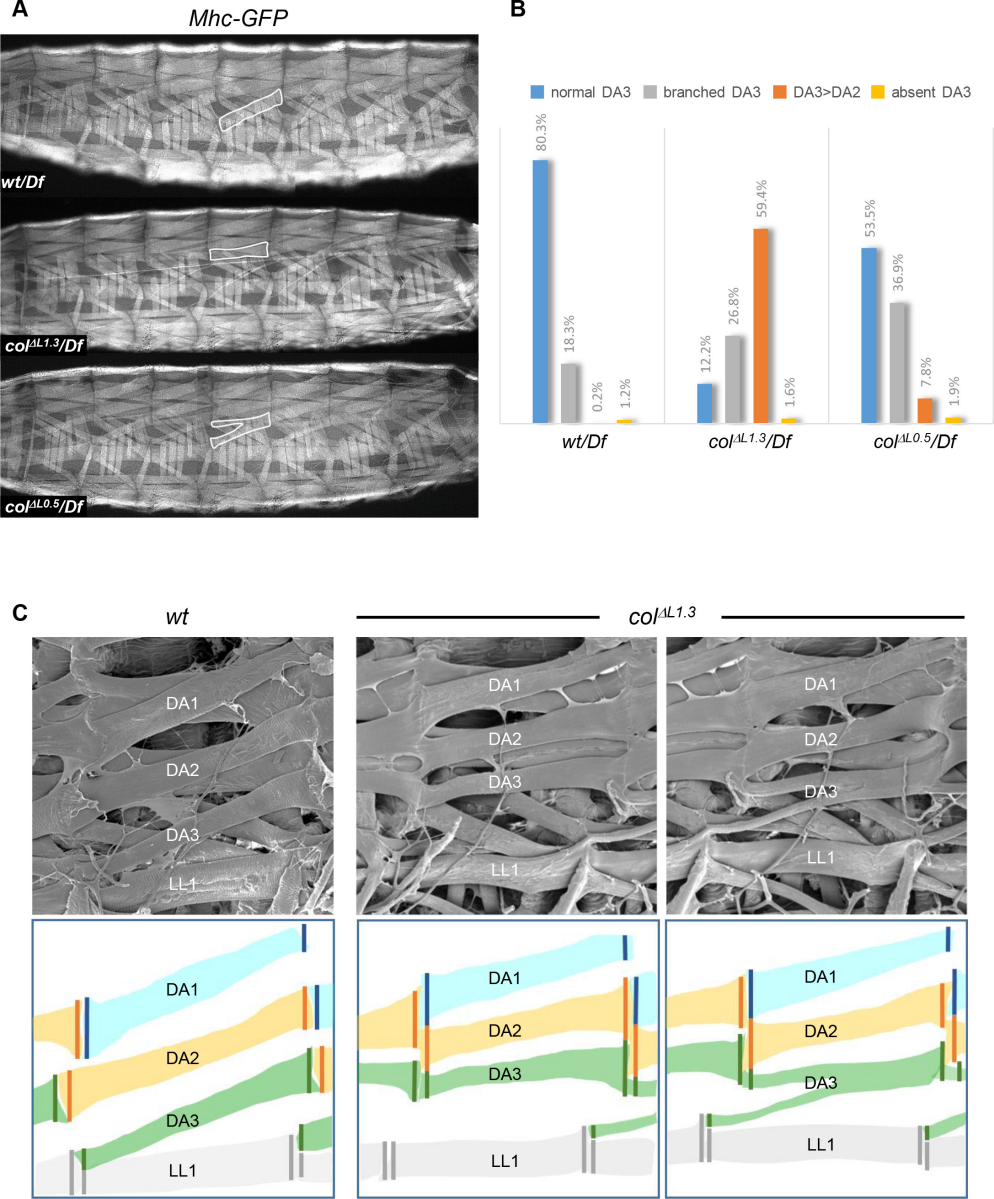
Muscle mismatching in L-CRM mutant larvae. (**A**) Mhc-GFP expression in control, *col^ΔL1.3^/Df* and *col^ΔL0.5^/Df* live 3^rd^ instar larvae; lateral views, anterior to the left. Examples of DA3, DA3^>DA2^ and DA3 branched muscles are circled in white. (**B**) Quantification of the relative proportions of DA3, branched DA3, DA3^>DA2^ and absence of DA3 muscles in A1 to A7 segments of *wt*/*Df*, *col^ΔL1.3^*/*Df* and *col^ΔL0.5^*/*Df;* Mhc-GFP larvae; (*wt/Df*, n = 375 segments/27 larvae; *col^ΔL1.3^*/*Df,* n = 320/23; *col^ΔL0.5^*/*Df*, n = 264/19). (**C**) Scanning electron microscopy of filleted larvae showing the dorsal and dorso-lateral muscles, schematically colour-coded below. In *wt* larvae, the DA muscles are parallel to each other within a segment with precise matching of the DA3/DA2 and DA2/DA1 MASs at each posterior segmental border. In *col^ΔL1.3^* larvae, DA3^>DA2^ muscles show homotypic, dorsal MASs and the DA3/LL1 connection is lost. The posterior MASs of branched DA3 is composite.

To investigate the impact of embryonic muscle patterning defects on *Drosophila* larval crawling, we first recorded the fraction of DA3, DA3^>DA2^ and branched muscles in 3^rd^ instar *wt* and *col^ΔLCRM^* larvae expressing GFP under control of the *Myosin heavy chain* (*Mhc*) promotor region (Figure 6A). It conforms to the statistics in late embryos (Figure 2B), except for an increased proportion of branched muscles, which could reflect under-evaluation of their number in embryos, due to threshold detection limit of L-CRM-moeGFP expression in thin fibres (Figure 6B). We then examined the pattern of MASs, using scanning electron microscopy (SEM) of dissected larval filets (Figure 6C; Figure 6—figure supplement 1). In *wt* larvae, as seen in stage 17 embryos (Figure 4A), the anterior edge of the DA3 muscle is anchored to a nodal lateral attachment site shared with the LL1 muscle, while its posterior edge aligns with the anterior edge of the DA2 muscle in the next posterior segment. Precise heterotypic DA2/DA1 iMASs are also visible over each intersegmental border. The regular alignment of the DA1, DA2 and DA3 muscles both draws strength lines spanning three adjacent segments, and regular tension surfaces between segments (Figure 6C; Figure 6—figure supplement 1). In *col^ΔL1.3^* larvae, the anterior DA3 muscle attachment has shifted from lateral to dorsal to form a DA3^>DA2^/DA3^>DA2^ homotypic iMAS (Figure 6C). This leads in turn to the formation of narrow, ectopic DA2/DA2 contacts and the DA1-DA2-DA3 strength line is distorted (Figure 6—figure supplement 1). In case of branched DA3 muscles, two strength lines co-exist (Figure 6C and Figure 6—figure supplement 1). In conclusion, SEM analyses of larval muscles show that loss of DA3 identity leads to ectopic iMASs between several dorsal internal muscles, distortion of the staggered ends architecture of DA muscles and of their alignment between consecutive segments.

**Figure 6—figure supplement 1.**
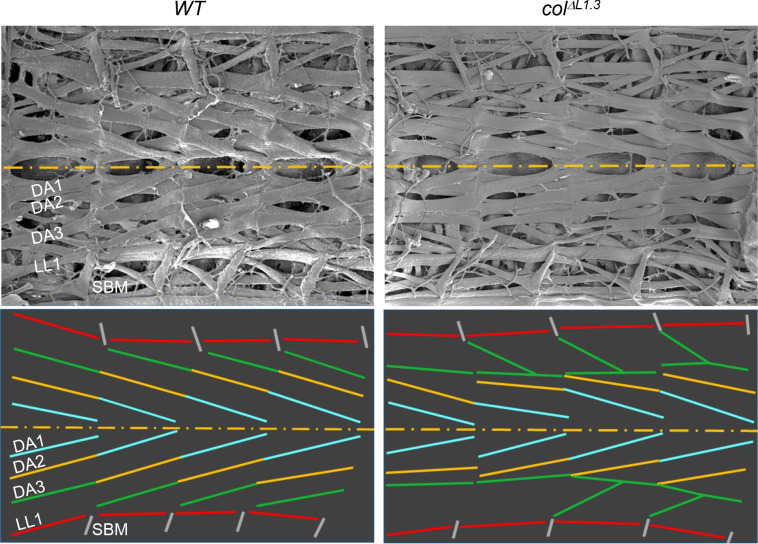
Scanning electron microscopy of wt and *col ^ΔL1.3^* internal muscles Larval fillets cut longitudinally along the ventral midline to expose the dorsal and dorso-lateral internal muscles. The dorsal midline is represented by a yellow dashed line. Upper panels: four abdominal segments of *wt* and *col ^ΔL1.3^* L3 larvae. Lower panels: muscle orientations are schematised by coloured lines, DA1 blue, DA2 yellow, DA3 green, LL1 red and SBM grey. In a wild type larva, the DA1, DA2 and DA3 muscles are oriented parallel and draw a force line over three consecutive segments and straight tension lines between two adjacent segments. In *col^ΔL1.3^* larvae, DA3^>DA2^ and branched DA3 transformations these lines are broken due to the formation of homotypic DA3^>DA2^ MAS and either loss or reduction of the DA3/LL1 connection.

### Branched muscles result in subtle locomotion defects

**Figure 7.**
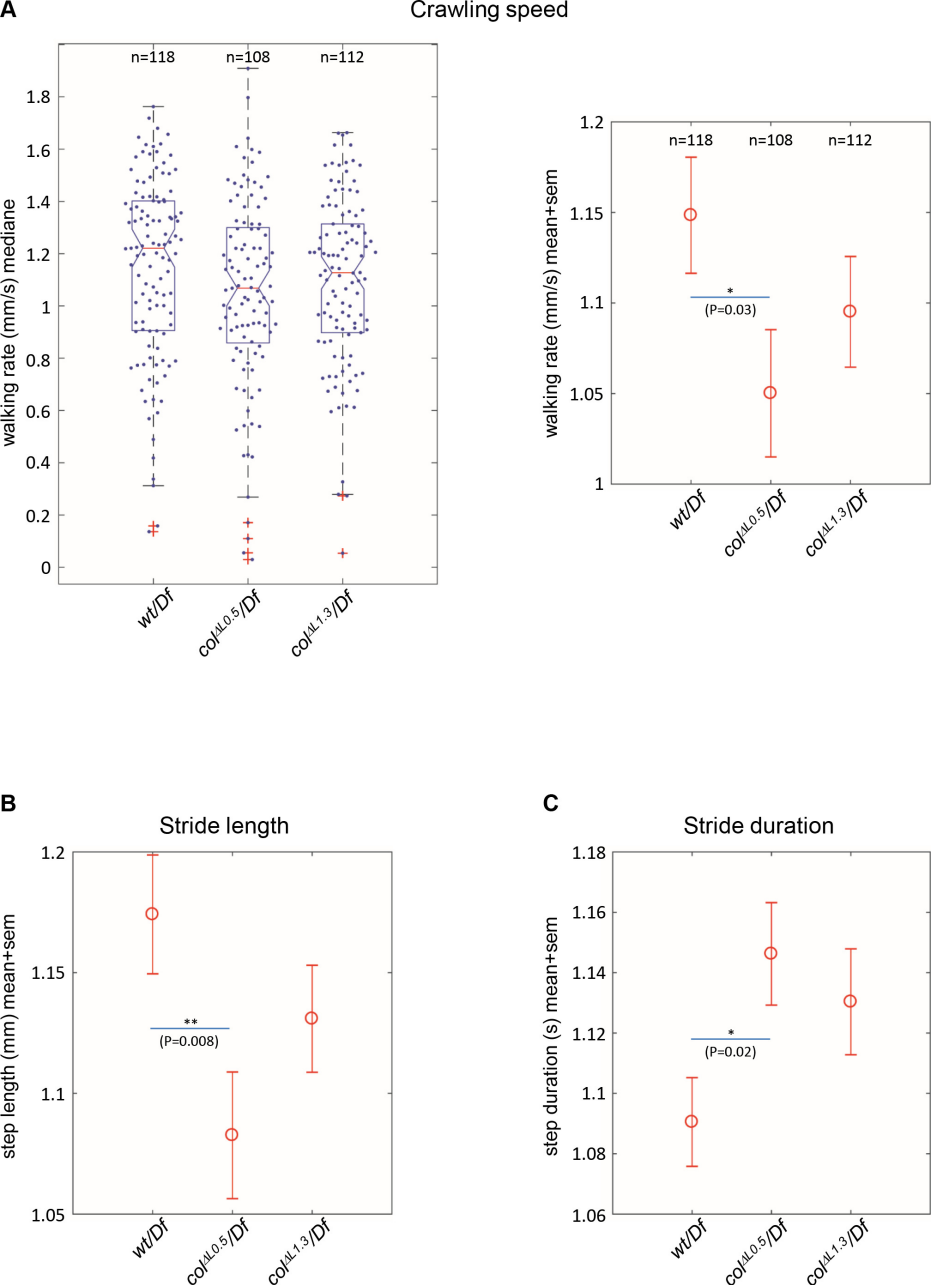
Branched muscles result in specific locomotion defects. (**A**) Left, Tukey’s diagrams (box-plot graph) showing the walking rate in mm/s (crawling speed) of *wt/Df* (n = 118), *col^ΔL0.5^/Df* (n = 108) and *col^ΔL1.3^*/*Df* (n = 112) larvae. Each point represents the average measurement for one larva, recorded during 20 s. 50% of points are located within the Tukey’s diagram. The red line gives the median, the narrowed area, the confidence interval of the median (95%). **Right**, same data as left, showing the mean speed ± standard error of mean (SEM) for each genotypes. (**B**) Stride length in mm. (**C**) Stride duration in s. The number of larvae (**n**) tested for each genotype is indicated in (**A**). Only significant differences are indicated (*p<0.05 and **p<0.01).

*Drosophila* larval crawling relies upon ordered abdominal body wall muscle contractions (Heckscher et al., 2012). The distorted muscle patterns observed in *col^ΔL1.3^* larvae raised the question of whether it impacted on locomotion. To address this question, we compared the locomotion of *+/Df, col^ΔL1.3^/Df* and *col^ΔL0.5^/Df* larvae using FIM (FTIR-based Imaging Method) and the FIMTrack software, which allows tracking simultaneously many larvae and quantitatively describing a variety of stereotypic movements (Risse et al., 2013; Risse et al., 2014; Risse et al., 2017). Here, we focused on three parameters: crawling speed, stride length, stride duration. We first recorded the ‘walking rate’, also called crawling speed, during the first 20 s, after larvae have been dropped on the agarose gel. At that time, larvae engage an ‘escape response’ corresponding to an active crawling phase (Figure 7A). Box-plot graphs (left) show intra-variability for each of the three genotypes. Beyond this variability, we observe, however, that *col^ΔL0.5^/Df* larvae display a significantly reduced crawling speed on average 1.05 ± 0.034 mm/sec (n = 108), compared to 1.15 ± 0.031 mm/sec for *+/Df* controls (n = 118), (p=0.03) (Figure 7A). To further investigate the origin of this speed reduction, we measured two crawling speed parameters: stride length and stride duration (Figure 7B–C). A significantly shorter stride was measured for *col^ΔL0.5^/Df* larvae (1.08 ± 0.025 mm) compared to control *+/Df* larvae (1.17 ± 0.024 mm), (p=0.008). Furthermore, stride duration was extended, from (1.09 ± 0.014 s) for *+/Df* larvae to 1.15 ± 0.017 s for *col^ΔL0.5^/Df* larvae. For both crawling speed, stride length and stride duration, *col^ΔL1.3^/Df* larvae (n = 112) display intermediate values. Yet, differences with either control or *col^ΔL1.3^*/*Df* larvae fall below the significance threshold level, suggesting that mis-orientation of the DA3^>DA2^ muscle does not, by itself, significantly impair the efficiency of segment contraction. Since larval crawling integrates information provided by neuronal networks, relayed by synaptic connections between motoneurons (MNs) and muscles, the mobility phenotype of *col^ΔL0.5^*/*Df* larvae could indicate defects motor innervation of DA3^>DA2^ muscles. The DA3 and DA2 muscles are innervated by the intersegmental nerve (ISN) which fasciculates motor axons reaching dorsal muscles (Hoang and Chiba, 2001; Landgraf and Thor, 2006). To examine the DA3^>DA2^ and branched DA3 innervation, we used anti-HRP and phalloidin staining to view ISN motoneuron projections and muscles, respectively (Figure 7—figure supplement 1). In *wt* embryos, the MN projection which innervates DA3 leaves the ISN ventral to the DA3 position and orients left such that the neuromuscular junction locates to the first anterior third of the muscle (100%; n = 28 segments). In case of a complete DA3^>DA2^ transformation, a MN projection is still observed (100%; n = 15 segments), which leaves the ISN at a more dorsal position than *wt*, reflecting the dorsal shift of the DA3^>DA2^, relative to DA3 muscle. In case of branched muscles, the lower branch is always innervated (100%, n = 30 segments). Only in 20% of the cases, the second, upper branch is also innervated.

**Figure 7—figure supplement 1.**
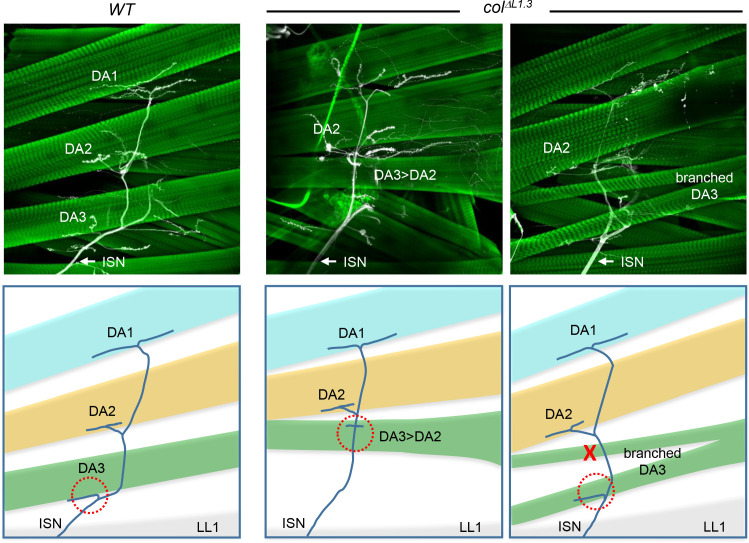
DA muscles innervation in L-CRM mutant larvae. Phalloidin and anti-HRP staining of *wt* and *col^ΔL1.3^* 3^rd^ instar larval fillets, showing internal DA muscles (green) and intersegmental nerve (ISN) projections (white), schematically colour-coded below. ISN projection to the DA3 muscle is circled by a red dashed line. Absence of projection is indicated by a red cross.

These innervation data indicate that fully transformed DA3 muscles are innervated, but only one branch of branched muscles is, in most cases. It remains to be established whether this asymmetric innervation contributes to the reduced crawling speed of *col^ΔL0.5^* larvae.

## Discussion

The stereotyped set of 30 somatic muscles in each abdominal segment which underlies *Drosophila* larval crawling has been thoroughly described many years ago (Bate, 1990; Bate and Rushton, 1993). One essential aspect laid out at that time was the concept of founder cell (FC), *i.e.*, the assignment to form a distinctive muscle to a single founder myoblast able to recruit other myoblasts by fusion. The morphological identity of each muscle is foreseen by a specific pattern of iTF expression in its FC (Tixier et al., 2010; Frasch, 1999). Here, we engineered a CRM deletion strategy connecting iTF expression to the larval musculature architecture and locomotion.

### iTF transcription in muscle PCs; redundant CRMs

Some iTFs are transiently transcribed during *Drosophila* muscle development, for example, *Kr* and *nau*, others such as *col*, *ladybird* and *slouch*/*S59*, at every step of the process (Dubois et al., 2016; Knirr et al., 1999; Bataillé et al., 2017; Dubois et al., 2007; Michelson et al., 1990; Jagla et al., 2002). Reporter analyses indicated that *col* transcription is controlled by two sequentially acting CRMs, of overlapping activity at the PC step, suggesting a handover mechanism between CRMs at this stage (Enriquez et al., 2012). However, deletion analyses show that L-CRM activity in PCs is not dependent, but redundant with E-CRM activity, and can be separated from *col* positive autoregulation that is specific to the DA3 lineage. This leads to a new model where iTF code refinement at each step of muscle identity specification, PMC >PC, PC >FC and FC >syncytial nuclei, is driven by a separate CRM. Distribution of the PC-identity information into two CRMs further supports the idea that iTF regulation at the PC stage is nodal to muscle identity specification (Carmena et al., 1998; Dubois et al., 2016; Enriquez et al., 2012; Jagla et al., 2002; Nose et al., 1998; Kumar et al., 2015). Our former analysis started to decrypt the combinatorial control of each dorso-lateral muscle identity, which involves at least eight different iTFs, in addition to Nau/MRF [Dubois et al., 2007]. Persistent, low level expression of Col protein in the DT1 and LL1 muscle precursors upon removal of the col autoregulatory module suggests the existence of both positively and negatively acting iTF-responsive elements in this module. Our present analysis concentrated on the DA3 lineage where *col* expression is maintained, whereas *col* is expressed in several PCs. Morphological transformations of other dorso-lateral muscles are observed at variable frequency in *col* protein null mutants (Enriquez et al., 2010), suggesting that E-CRM activity provides robustness to the combinatorial control of identity of these muscles and that robustness is likely also contributed by other iTFs (Dubois et al., 2016; Schnorrer and Dickson, 2004). PC/FC specific CRMs have only been functionally identified for a handful of muscle iTFs (Rivera et al., 2019). Computational predictions identified, however, several thousand putative muscle enhancers and uncovered extensive heterogeneity among the combinations of transcription factor binding sites in validated enhancers, beside sites for the core intrinsic muscle regulators Tin, Mef2 and Twi (Sandmann et al., 2007; Gisselbrecht et al., 2013; Cusanovich et al., 2018). Dissecting whether step-specific and redundant/distributed CRM configurations (Cannavò et al., 2016; Hong et al., 2008; Frankel et al., 2010) apply to many muscle iTFs and underlie the progressively refined control of final muscle patterns is a future step.

### Distinctive muscle morphology requires identity reprogramming of fused myoblasts

The process by which iTFs determine the final morphological features of each muscle, is not fully understood. Fusing FCM nuclei generally adopt the iTF protein code of the FC nucleus, while propagation of iTF transcription and activation of realisation genes is muscle lineage-specific (Boukhatmi et al., 2012; Crozatier and Vincent, 1999; Knirr et al., 1999; Bataillé et al., 2010; Bataillé et al., 2017; Bourgouin et al., 1992). We have previously shown that Col protein import precedes activation of *col* transcription in fused FCM nuclei, and correlates with the activation of realisation genes, a sequence of events termed syncytial identity reprogramming (Bataillé et al., 2017; Dubois et al., 2007). Upon loss of *col* transcription in the DA3 FC (*col^Δ1.3^* embryos), there is a complete DA3^>DA2^ transformation. When *col* transcription is maintained (*col^ΔL0.5^* embryos), in the DA3 FC but not propagated to other syncytial nuclei, there is an incomplete DA3 transformation into branched muscles. From this, two conclusions can be drawn: 1) Final selection of myotendinous connection sites is intrinsic to FC identity. 2) Identity reprogramming of syncytial nuclei is required for robustness of this selection and precise muscle patterns.

### Identity shifts, a source of branched muscles

Live imaging of lateral-oblique and ventral transverse muscles development distinguished 3 phases of muscle elongation (Schnorrer and Dickson, 2004): 1) FC migration, stage 12, ending with the first FC/FCM fusion event and stretching of the muscle precursor along a given axis; 2) bipolar myotube elongation, characterised by the presence of extensive filopodia at both axis ends, in search for attachment sites, stages 13 to 15; 3) maturation of myotendinous attachment, stage 16. The formation of DA3^>DA2^ and branched muscles in CRM mutants recalls a transient exploration by the wt DA3 muscle precursor of both dorsal and lateral tendon cells, a process deviating from the bipolar migration/attachment scheme (Schweitzer et al., 2010; Enriquez et al., 2012; Schnorrer and Dickson, 2004; Bahri et al., 2009). Interestingly, ectopic Col expression in the DA2 muscle leads to reciprocal DA2^>DA3^ transformation as well as branched muscles (Boukhatmi et al., 2012). This indicates that selection of dorsal *versus* lateral tendon cells is a highly controlled process. A few molecules involved in targeted attachment of subsets of muscles to specific tendon cells have been identified: Kon tiki/Perdido, a single pass transmembrane protein and the PDZ protein DGrip for proper elongation of ventral longitudinal muscles (Schnorrer et al., 2007); the ArfGAp protein Git for sensing integrin signaling and halting elongation of Lateral Transverse (LT) muscles once their attachment site has been reached (Bahri et al., 2009; Richier et al., 2018). Robo/Slit signaling attracts muscles at segmental borders, the Slit ligand being expressed by tendons, and Robo and Robo2 receptors by elongating muscles. slit also acts as a short-range repellent contributing to the collapse of leading-edge filopodia when a muscle reaches the tendon extracellular matrix (Ordan and Volk, 2015; Ordan et al., 2015). In vivo imaging showed that the DA3 muscle fails to stop at the segment border in slit mutants and sometimes branches (Ordan et al., 2015). However, this is not observed in *col^ΔLCRM^*; the DA3 slit branching pattern is rather similar to that frequently observed in nau mutants, with two posterior attachment sites in place of one (Dubois et al., 2016; Boukhatmi et al., 2012). Which realisation genes downstream of iTFs are responsible for the precision of DA attachment sites, that is, proper balancing attraction/repulsion cues, should be the focus of future studies.

### Muscle staggered ends; heterotypic *versus* homotypic interactions?

In addition to attaching to tendon cells, it was previously shown that internal muscles attach to each other (Maartens and Brown, 2015; Bate and Rushton, 1993). Detailed imaging in both embryos and larvae shows that the DA3/DA2 and DA2/DA1 attachment sites precisely match over each segmental border, such that larval DA1, DA2, and DA3 align over three consecutive segments. Recording DA3 and DA3^>DA2^ muscle development by a combination of live imaging and immunostainings shows that DA3/DA2 matching in wt embryos results from both heterotypic DA3/DA2 attraction and, possibly, homotypic repulsion. Prior attachment of the posterior DA3 edge to dorsal tendon cells leads to retraction of filopodia issued from the homologous muscle in the next adjacent segment while stabilisation of heterotypic contacts results into DA3/DA2 iMAS formation over the intersegmental border. A similar process results in alignment of DA2 with DA1. Interestingly, DA3^>DA2^ establish both homotypic iMASs, and heterotypic iMASs with DA2, suggesting preserved attraction to tendon cells and partial loss of preferential heterotypic adhesion. Prior posterior attachment was previously observed during development of abdominal adult muscles (Currie and Bate, 1991). Whether this temporal sequence is instrumental in the precise matching of muscles over each segmental border and whether competition between muscles for the same tendon cells is also involved remain to be assessed. Cell matching is a widely used process during embryogenesis to construct complex tissue architecture. Selective filopodia adhesion has recently been shown to ensure precise matching between identical cardioblasts and boundaries between different cell identities in the *Drosophila* heart. In this case, homotypic matching is linked to differential expression by each cell type of the adhesion molecules, Fasciclin III and Ten-m (Zhang et al., 2018). Transcriptome analyses of specific muscles at different developmental times should allow to identify attractive and, possibly, repulsive molecules, acting in muscle precise matching.

### Branched muscles impact on crawling speed

*Drosophila* larval crawling is a well-suited paradigm to link muscle contraction patterns and locomotor behaviour. Longitudinal, acute and oblique muscles within a larval segment contract together and, as they begin to relax, the contraction is propagated to the next segment, creating a peristaltic wave from tail to head (forward locomotion), or head to tail (backward locomotion) (Heckscher et al., 2012). The rhythmic movements of locomotion are part of behavorial routines that facilitate the exploration of an environment. Exploratory routines alternate straight line movement also called ‘active crawling phase’, with change of direction, the ‘reorientation phase’ (Günther et al., 2016; Berni et al., 2012; Lahiri et al., 2011). The active larval crawling phase requires an intense, prolonged muscular effort. In this study, we focused on crawling parameters during this phase. The crawling speed during the escape response is not significantly reduced in *col^ΔL1.3^*/*Df*, compared to control +/*Df* larvae, indicating that muscle contraction is properly controlled and that a mechanical compensation mechanism for the DA3 mis-orientation could occur. However, it is significantly reduced in *col^ΔL0.5^*/*Df* larvae. This seems paradoxical because the number of DA3^>DA2^ transformed muscles is higher in *col^ΔL1.3^*/*Df* larvae. *col^ΔL0.5^*/*Df* larvae present many branched muscles, however. While DA3^>DA2^ are always innervated, only one branch of branched muscles is, most of the time, raising the possibility that branched muscles do not contract properly, or with a gap in time (see Zarin et al., 2019). From these different observations, we can conclude: i) single muscle transformations only moderately impact crawling speed, raising the possibility of biomechanical compensation by other muscles; ii) branched muscles could be less efficient than fully transformed muscles. At this point, the reason why, – either mechanic weakness, improper innervation or impaired Ca^2+^ wave propagation, antagonistic force lines upon muscle contraction - may only be object of speculation.

The generation of branched muscles in *Drosophila* identity mutants is one important finding as branched muscle fibres accumulate in humans, following muscle regeneration after damage or in Duchenne muscular dystrophy patients (Chan and Head, 2011). It opens the possibility to investigate, in vivo, how physiological properties of branched fibres differ from morphologically normal fibres and associated mechanical instability in an otherwise normal muscle pattern.

## Materials and methods

**Key resources table:** 

Reagent type (species) or resource	Designation	Source or reference	Identifiers	Additional information
Strain, strain background (*Drosophila melanogaster*)	white^[1118]^	Bloomington *Drosophila* Stock Center	BDSC Cat# 3605, RRID:BDSC_3605	
Strain, strain background (*Drosophila melanogaster*)	Df(2L)BSC429	Bloomington *Drosophila* Stock Center	BDSC Cat# 24933, RRID:BDSC_24933	
Strain, strain background (*Drosophila melanogaster*)	vasa-cas9VK00027	Bloomington *Drosophila* Stock Center	BDSC Cat# 51324, RRID:BDSC_51324	
Strain, strain background (*Drosophila melanogaster*)	sr-Gal4			obtained from G. Morata, Madrid, Spain
Strain, strain background (*Drosophila melanogaster*)	Col^1^	Our lab		Crozatier and Vincent, 1999
Strain, strain background (*Drosophila melanogaster*)	Mi{MIC}knMI15480/SM6a	Bloomington *Drosophila* Stock Center	BDSC Cat# 67516, RRID:BDSC_67516	MiMic Line
Strain, strain background (*Drosophila melanogaster*)	GMR69G03	Bloomington *Drosophila* Stock Center	BDSC Cat# 39500, RRID:BDSC_39500	GMR line, GMR located in the Vg gene
Strain, strain background (*Drosophila melanogaster*)	GMR69G04	Bloomington *Drosophila* Stock Center	#46616	GMR line, GMR in the Vg gene
Strain, strain background (*Drosophila melanogaster*)	GMR69G05	Bloomington *Drosophila* Stock Center	#39501	GMR line, GMR in the Vg gene
Strain, strain background (*Drosophila melanogaster*)	GMR69G06	Bloomington *Drosophila* Stock Center	#39502	GMR line, GMR in the Vg gene
Strain, strain background (*Drosophila melanogaster*)	GMR69G07	Bloomington *Drosophila* Stock Center	BDSC Cat# 47956, RRID:BDSC_47956	GMR line, GMR in the Vg gene
Strain, strain background (*Drosophila melanogaster*)	GMR69G08	Bloomington *Drosophila* Stock Center	BDSC Cat# 46617, RRID:BDSC_46617	GMR line, GMR in the Vg gene
Strain, strain background (*Drosophila melanogaster*)	GMR69G09	Bloomington *Drosophila* Stock Center	BDSC Cat# 46618	GMR line, GMR in the Vg gene
Strain, strain background (*Drosophila melanogaster*)	GMR69G10	Bloomington *Drosophila* Stock Center	BDSC Cat# 39503	GMR line, GMR in the Vg gene
Strain, strain background (*Drosophila melanogaster*)	GMR69G12	Bloomington *Drosophila* Stock Center	BDSC Cat# 46619	GMR line, GMR in the Vg gene
Strain, strain background (*Drosophila melanogaster*)	GMR69G03	Bloomington *Drosophila* Stock Center	BDSC Cat# 46620, RRID:BDSC_46620	GMR line, GMR in the Vg gene
Strain, strain background (*Drosophila melanogaster*)	GMR12A09	Bloomington *Drosophila* Stock Center	BDSC Cat# 47319	GMR line, GMR in the Kn gene
Strain, strain background (*Drosophila melanogaster*)	GMR12G07	Bloomington *Drosophila* Stock Center	BDSC Cat# 47854, RRID:BDSC_47854	GMR line, GMR in the Kn gene
Strain, strain background (*Drosophila melanogaster*)	GMR12H01	Bloomington *Drosophila* Stock Center	BDSC Cat# 48528, RRID:BDSC_48528	GMR line, GMR in the Kn gene
Strain, strain background (*Drosophila melanogaster*)	GMR13A11	Bloomington *Drosophila* Stock Center	BDSC Cat# 49248	GMR line, GMR in the Kn gene
Strain, strain background (*Drosophila melanogaster*)	GMR13B06	Bloomington *Drosophila* Stock Center	BDSC Cat# 48544, RRID:BDSC_48544	GMR line, GMR in the Kn gene
Strain, strain background (*Drosophila melanogaster*)	GMR13B08	Bloomington *Drosophila* Stock Center	BDSC Cat# 48546, RRID:BDSC_48546	GMR line, GMR in the Kn gene
Strain, strain background (*Drosophila melanogaster*)	GMR13C09	Bloomington *Drosophila* Stock Center	BDSC Cat# 48555, RRID:BDSC_48555	GMR line, GMR in the Kn gene
Strain, strain background (*Drosophila melanogaster*)	GMR13C11	Bloomington *Drosophila* Stock Center	BDSC Cat# 48556, RRID:BDSC_48556	GMR line, GMR in the Kn gene
Strain, strain background (*Drosophila melanogaster*)	GMR13F08	Bloomington *Drosophila* Stock Center	BDSC Cat# 48576, RRID:BDSC_48576	GMR line, GMR in the Kn gene
Strain, strain background (*Drosophila melanogaster*)	GMR13F10	Bloomington *Drosophila* Stock Center	BDSC Cat# 48578, RRID:BDSC_48578	GMR line, GMR in the Kn gene
Strain, strain background (*Drosophila melanogaster*)	GMR47D05	Bloomington *Drosophila* Stock Center	BDSC Cat# 47605, RRID:BDSC_47605	GMR line, GMR in the Kn gene
Strain, strain background (*Drosophila melanogaster*)	GMR46H09	Bloomington *Drosophila* Stock Center	BDSC Cat# 54712, RRID:BDSC_54712	GMR line, GMR in the Kn gene
Antibody	anti-col (Mouse monoclonal)	Our lab		1:50 Krzemień et al., 2007
Antibody	anti-LacZ (Mouse monoclonal)	Promega	Promega Cat# Z3781, RRID:AB_430877	1:1000
Antibody	anti-spectrin (Mouse monoclonal)	Hybridoma Bank	DSHB Cat# 3A9 (323 or M10-2), RRID:AB_528473	1:200
Antibody	anti-GFP (Rabbit polyclonal)	Biolabs	Torrey Pines Biolabs Cat# TP401 071519, RRID:AB_10013661	1:1000
Antibody	anti-GFP (Chicken polyclonal)	Abcam	Abcam Cat# ab13970, RRID:AB_300798	1:500
Antibody	Phalloidin-texas red	Thermofisher Scientific	Cat# Cat#T7471	1:500
Antibody	Alexa fluor antibodies 488, 555 and 647	Molecular probes		1:300
Antibody	Alexa fluor 594 anti HRP	Jackson Immunological research	Jackson ImmunoResearch Labs Cat# 123-585-021, RRID:AB_2338966	1:300
Antibody	Alexa fluor phalloidin	Thermofisher Scientific	Thermo Fisher Scientific Cat# A12381, RRID:AB_2315633	1:500
Antibody	Biotinylated goat anti-mouse	Vector Laboratories	Vector Laboratories Cat# BA-9200, RRID:AB_2336171	1:2000
Software, algorithm	ImageJ	Schneider et al., 2012	ImageJ, RRID:SCR_003070	https://imagej.nih.gov/ij/
Software, algorithm	FIMTrack	Risse et al., 2014		https://www.uni-muenster.de/PRIA/en/FIM/
Software, algorithm	MATLAB	MathWorks	MATLAB, RRID:SCR_001622	https://www.mathworks.com/products/matlab-online.html
Other	FISH probes labelled with Quasar dye 670 (col)	Biosearch Technologies and this study		Kn first intron
Other	FISH probes labelled with Quasar dye 570 (nau)	Biosearch Technologies and this study		Nau first and third introns

### Fly strains

All *Drosophila melanogaster* stocks and genetic crosses were grown using standard medium at 25°C. The strains used were *white^[1118]^, col*LCRM 4–0.9 (Enriquez et al., 2010), *col^1^* (Crozatier and Vincent, 1999), *sr-Gal4* (obtained from G. Morata, Madrid, Spain). The 12 *kn* and 10 *vg* Janelia-Gal4 lines (GMR) (Pfeiffer et al., 2008), *UAS-mcd8RFP, Mhc-GFP*, *Df(2L)BSC429*, kn^MI15480^ y^1^ w^*^; Mi{MIC}kn^MI15480^/SM6a (BDSC_67516) (Nagarkar-Jaiswal et al., 2015)*, vasa-cas9^VK00027^* (BDSC_51324), *Ilk-GFP* (*w^1118^*; *P{PTT-GB}Ilk^ZCL3111^*) (BDSC_6831), lines were provided by the Bloomington *Drosophila* Stock Center. The *col^1^* and Df(2L)BSC429 strains were balanced using CyO,{*wg^en11^-lacZ*} or CyO,{*dfd-YFP*} and homozygous mutant embryos or larvae identified by absence of lacZ or YFP expression, respectively.

### CRM deletions generated by Crispr/Cas9

Genomic col target sites were identified using http://tools.flycrispr.molbio.wisc.edu/targetFinder/ (Gratz et al., 2014). Prior to final selection of RNA guides (gRNA) for deletions of *col* CRMs, genomic PCR and sequencing of DNA from *kn^MI15480^* and *vasa-cas9^VK00027^* flies was performed to check for polymorphisms in the targeted regions. Guides targeting E-CRM and L-CRM were inserted in the pCFD4: U6:3-gRNA vector (Addgene n^o^: 49411) as described (Port et al., 2014); (see http://www.crisprflydesign.org/wp-content/uploads/2014/06/Cloning-with-pCFD4.pdf). All guides were verified by sequencing. The sequences of the oligonucleotides used to construct each gRNA expression plasmid are given in Figure 1—figure supplements 1 and 2. To delete the core region of L-CRM*, vasa-cas9* embryos were microinjected with gRNAs in pCFD4 (200 ng/μl). To delete the E-CRM, *kn^MI15480^* embryos were injected with gRNA in pCFD4 (150 ng/μl) and pAct-Cas9U6 (400 ng/μl). Each adult hatched from an injected embryo was crossed to the balancer stock *sna^Sco^/*CyO, {*wg^en11^-LacZ*} and 100–200 F1 fly were individually tested for either *col* CRM deletion by PCR on genomic DNA. A pre-screening for E-CRM deletion was based on the loss of *yellow* carried by Mimic *kn^MI15480^*.

### Reporter constructs

The *yellow* intron (*yi*), FlyBase ID #FBgn0004034 (position: 356918–359616) was inserted in the *lacZ* coding region between aa (Tyr 952) and aa (Ser 953) by standard PCR-based cloning position. The resulting fragment was cloned downstream of L-CRM inserted in a pAttB vector, and micro-injected in embryos for chromosomal insertion at position 68A4. VgM1-moeGFP was constructed by PCR amplification of the GMR69G04 and GMR69G05 overlap. The 1.4 kb amplicon (named VgM1) was inserted upstream of moeGFP to generate the pAttB VgM1-moeGFP construct. It was inserted at position 68A4 on the third chromosome.

### Immunohistochemistry

Antibody staining, in situ hybridisation with intronic probes and phalloidin staining were as described previously (Dubois et al., 2007). Primary antibodies were: mouse anti-Col (1/50; Boukhatmi et al., 2012; Dubois et al., 2007), anti-LacZ (1/1000; Promega), mouse anti α-Spectrin (1/200; Hybridoma Bank), rabbit anti-GFP (1/1000; Torrey Pines Biolabs), chicken anti-GFP (1/500; Abcam), Phalloidin-Texas RedX (1/500; Thermofisher Scientific). Secondary antibodies were: Alexa Fluor 488-, 555- and 647- conjugated antibodies (1/300; Molecular Probes) and biotinylated goat anti-mouse (1/2000; Vector Laboratories).

### Motoneurons and muscles visualisation

To both visualise motoneuron axonal pathways and muscles, fillets of control and *col^ΔL1.3^* third instar larvae were incubated overnight with Alexa 594-conjugated anti-HRP (1/300; Jackson Immunological Research) and Alexa Fluor 488 Phalloidin (1/500; Thermofisher Scientific), at 4°C. To prepare fillets, third instar larvae placed in myorelaxant buffer (Yalgin et al., 2011), were cut longitudinally on the ventral side to expose the dorsal and dorso-lateral musculature. Fillets were then fixed 1 hr in 4% formaldehyde and washed in PBT.

### In situ hybridisation

In situ hybridisation with Stellaris RNA FISH probes were done as described by the manufacturer for *Drosophila* embryos (https://www.biosearchtech.com). The FISH probe sets for *col* and *nau* were designed using the Stellaris probe designer (https://www.biosearchtech.com/stellarisdesigner) and labelled with Quasar 670 Dye (*col*) and Quasar 570 Dye (*nau*) (Stellaris Biosearch Technologies). One set of 48 oligonucleotides was designed against the first *col* intron to detect primary nuclear transcripts. Another set of 48 oligonucleotides was also designed against the first and third *nau* introns. When antibody staining and FISH were combined, the standard immuno-histochemistry protocol was performed first, with 1 U/μl of RNase inhibitor from Promega included in all solutions, followed by the FISH protocol. Confocal sections were acquired on Leica SP8 or SPE microscopes at 40x or 63x magnification, 1024/1024 pixel resolution. Images were assembled using ImageJ and Photoshop softwares.

### Quantification of *col* transcription

To quantify the level of *col* nuclear transcripts in FCs and PCs, we calculated the ratio between *col* and *nau* hybridisation signals using intronic probes. Before using *nau* as internal reference we verified that *nau* transcription level is not modified in *col* L-CRM mutants. The same laser parameters were set for all intronic probes and at least five different embryos at each stage 11 and 12 were recorded. Optimal Z stacks were acquired at × 40. ImageJ was used to analyse the data. For each stack, a Sum slices projection was generated. Each region of interest (ROI), corresponding to a DA3 nucleus, was manually drawn, based on Mef-2 immunostaining. The same ROI served to determine the intensity of *nau* and *col* signals on the green and red channels, respectively (Figure 3—figure supplement 2A). A threshold was applied to each channel to remove background. Data plots and statistical analyses were performed with Prism 5.0 using unpaired *t*-test.

### Phenotype quantification at embryonic and larval stages

To quantify embryonic phenotypes, L-CRM-moeGFP embryos were immunostained with a primary mouse anti-GFP (1/500) (Roche) and secondary biotinylated goat anti-mouse (1/2000) (VECTASTAIN ABC Kit). Stained embryos were imaged using a Nikon eclipse 80i microscope and a Nikon digital camera DXM 1200C. A minimum 100 A1-A7 abdominal segments of stage 15–16 embryos were analysed for each genotype. (+/Df: n = 127 segments - 16 embryos; *col^ΔE^*/Df: n = 170 segments - 23 embryos; *col^ΔL1.3^*/Df: n = 190 segments - 27 embryos; *col^ΔL0.5^*/Df: n = 103 segments - 13 embryos). To quantify larval phenotypes, wandering L3 larva displaying *Mhc-GFP* reporter line were immobilised between slide and coverslip, and left and right larval sides imaged using Nikon AZ100 Macroscope at 5x magnification. Minimum 260 abdominal segments were analysed for each genotype. (+/Df: n = 375 segments - 27 larvae; *col^ΔL1.3^*/Df: n = 320 segments - 23 larvae; *col^ΔL0.5^*/Df: n = 264 segments - 19 larvae).

### Live imaging embryonic muscle development

Embryos were bleach dechorionated and stage 12 embryos manually picked, laterally orientated and mounted on a coverslip coated with heptane glue to prevent drift during imaging. A drop of water was placed on the embryos to maintain their survival. Images were collected on a Leica TCS-SP8 confocal using a 25X water immersion lens. Sections were recorded every 130 to 150 s for the wt embryos and every 120 to 160 s for the *col^ΔL1.3^* embryos, and z-stacks collected with optical sections at maximum 1 µm interval. Image processing was performed with Fiji (http://fiji.sc/wiki/index.php/Fiji) and custom programming scripts in Fiji. The z-stacks projections were corrected in x and y dimensions by manual registration using a reference point tracking.

### Scanning electron microscopy (SEM)

To prepare fillets, third instar wild type and homozygous *col^ΔL1.3^* larvae raised at 25°C were dissected in myorelaxant buffer, according to Gratz et al., 2014. Larvae were cut longitudinally on the ventral side to preserve and expose the dorsal and dorso-lateral musculature. Fillets were then fixed 1 hr in a 4% formaldehyde/2.5% glutaraldehyde mixture in 1X PBS, washed in water and dehydrated gradually in ethanol. Fillets were dried at the critical point (Leica EM CPD 300 critical point apparatus), covered with a platinum layer (Leica EM MED 020 metalliser) and imaged with a Quanta 250 FEG FEI scanning microscope.

### Behavioral analysis

We conducted locomotion assays by tracking the trajectory of larvae using the FIM method (Risse et al., 2013). Wandering third instar larvae were gently picked up with a paintbrush and transferred to an agar plate. The larvae were then videotaped using a digital camera (Baumer VCXG53M); lentille (Kowa LM16HC); infrared filter (IF093SH35.5). Each video containing 5 to 10 larvae per run, on a 1% agarose gel, was recorded at five frames/sec for 20 s. Individual larva were tracked using the FIMTrack software (Risse et al., 2014), which provided the position across time of five points regularly spaced along the spine of each animal, from head to tail. Analysis was done by using MATLAB software. Peristalsis cycles were obtained using the derivative of the spine length (i.e., sum of the distance across successive point along the spine) through time, which provide a time series smoothly oscillating around zero. For each peristalsis cycle, we measured Stride length (centroid displacement across each cycle) and Stride duration (cycle duration). Walking rate was obtained by measuring the distance of the centroid (3^rd^ spine point) across successive frame. These values were averaged for each individual across the 20 s of recording. Statistical comparisons between genotypes were computed using a linear model with GenoT as fixed effect, and individual larva as a statistical unit.

## Data Availability

All data generated or analysed during this study are included in the manuscript and supporting files.

## Additional files

Transparent reporting form
